# Comprehensive identifying flavonoids in *Citri Reticulatae Pericarpium* using a novel strategy based on precursor ions locked and targeted MS/MS analysis

**DOI:** 10.1038/s41598-024-60415-w

**Published:** 2024-04-27

**Authors:** Hong-Ping Wang, Zhao-Zhou Lin, Hui Wang, Xuan Yang, Nan Niu

**Affiliations:** 1 Scientific Research Institute of Beijing Tongrentang Co., Ltd., Beijing, 100011 China; 2 Beijing Zhongyan Tongrentang Pharmaceutical R & D Co., Ltd., National Engineering Research Center for R&D of TCM Multi-Ingredient Drugs, Beijing, 100079 China

**Keywords:** *Citri Reticulatae Pericarpium*, Flavonoids, Locked, Targeted MS/MS analysis, Plant sciences, Secondary metabolism

## Abstract

*Citri Reticulatae Pericarpium* is a traditional Chinese medicine with extremely high health benefits as well as clinical value. In vivo and in vitro tests have proved that its main active secondary metabolites are flavonoids. However, they have not been comprehensively analyzed up to now mainly due to lack of suitable analysis method. To solve this problem, a novel strategy based on precursor ions locked and targeted MS/MS analysis was proposed. Firstly, the database of the flavonoids previously isolated from *Citri Reticulatae Pericarpium* was established to obtain the characteristics of their precursor ions. Secondly, after performing the full MS scan of the extract, all compounds in the total ion chromatogram were extracted by Compound Discoverer software. Thirdly, the precursor ions of the flavonoids were locked from the extracted compounds according to their characteristics, forming a precursor ions list. Finally, the precursor ions in the constructed list were performed targeted MS/MS analysis for structures characterization. As a result, total 187 flavonoids were successfully identified, and except for flavones, flavonols as well as dihydroflavones, some chalcones were also characterized from *Citri Reticulatae Pericarpium* for the first time.

## Introduction

As a commonly used traditional Chinese medicine in China, *Citri Reticulatae Pericarpium*, which is the dried matured pericarp of *Citrus reticulata Blanco* as well as its cultivated varieties belonging to rutaceae family, is widely used in clinical practice. And except for its medicinal value with abundant resources, *Citrus reticulata Blanco* is also an edible material with highly exploitable value. Modern studies correlated to its pharmacological actions, biological activities, and clinical applications have revealed that treatment of cardiovascular disease^1^, anti-oxidant^2^, anti-inflammatory^3^, anti-tumor^4^ as well as reducing blood lipid are its important effects^5^. However, with the development of evidence-based medicine and/or evidence-based pharmacy, clarification of the active secondary metabolites in *Citri Reticulatae Pericarpium* is the fundamental science, which needs to be systematically addressed. In vivo and in vitro tests have revealed that the above mentioned effects are all related to flavonoids, a group of chemical substances which are regarded as the characteristic constituents of *Citri Reticulatae Pericarpium*^1,6–9^, needing to be comprehensively analyzed.

In fact, there are relatively few studies on the secondary metabolites of *Citri Reticulatae Pericarpium*, and from the small amount of published articles, we found that there are two approaches for analysis of secondary metabolites in *Citri Reticulatae Pericarpium*, mainly including traditional chemical separation method and liquid chromatography-mass spectrometry (LC–MS) method based on modern technology. In the chemical separation method, the extract of *Citri Reticulatae Pericarpium* was subjected to silica gel column chromatography, targeting acquisition of the monomers of secondary metabolites^10,11^. However, some secondary metabolites were lost during the progress of separation, and usually secondary metabolites with high amounts were obtained leading to so far 41 flavonoids were isolated from *Citri Reticulatae Pericarpium* and *Citri Reticulatae Pericarpium Viride*^12^. In the LC–MS method, ultra performance liquid chromatography (UPLC) combined with high resolution mass spectrometry (HR MS) was commonly used to detect the secondary metabolites in *Citri Reticulatae Pericarpium*. Relatively to the chemical separation method, much more secondary metabolites were analyzed. For example, total 32–73 secondary metabolites were identified from *Citri Reticulatae Pericarpium* with the help of ultra performance liquid chromatography-quadrupole-time of flight mass spectrometry (UPLC-Q-TOF-MS)^13–16^, and 61 secondary metabolites were characterized from *Citri Reticulatae Pericarpium* using ultra performance liquid chromatography-ion trap/time of flight mass spectrometry (UPLC-IT-TOF-MS)^17^. Although UPLC-HR MS is an extremely effective means for secondary metabolites analysis, the current identified secondary metabolites are still far less than those in *Citri Reticulatae Pericarpium*, mainly due to lacking of a method of acquiring the fragmentation ions of much more secondary metabolites.

Generally, sufficient fragmentation ions are crucial for characterization of a secondary metabolite, and one of the important prerequisites for obtaining sufficient fragmentation ions of a secondary metabolite is that its precursor ion is selected and fragmented during MS/MS analysis. The aim of the study is to systematically identify the flavonoids, which are the main secondary metabolites in *Citri Reticulatae Pericarpium*. And thus, a strategy based on their precursor ions locked and targeted MS/MS analysis was proposed. Using this strategy, a total of 187 flavonoids were successfully characterized. The number of the identified flavonoids was improved greatly comparing to that previously reported. In addition, the analysis strategy established in our study provides a reference for analysis and identification of flavonoids in other traditional Chinese medicines.

## Results and discussion

### The established analysis strategy

**Figure 1:**
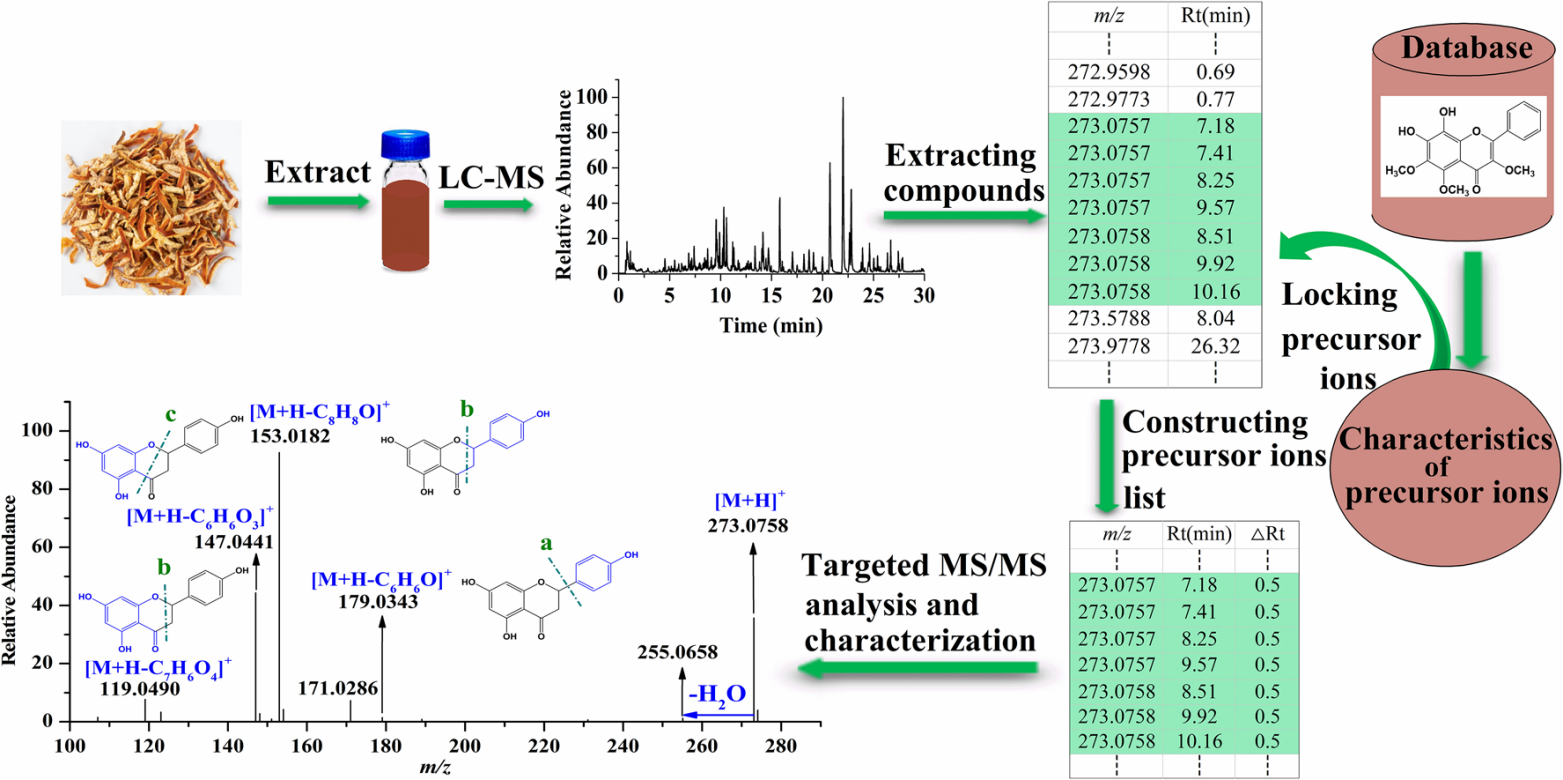
The established strategy based on precursor ions locked and targeted MS/MS analysis.

The established analysis strategy (shown in Fig. 1) mainly included the following four steps:

1.Establishing the database of the known flavonoids to obtain the characteristics of their precursor ions.

Generally, chemical components belonging to the same chemical cluster in a traditional Chiniese medicine own similar chemical structures and molecular characteristics. Based on this, the flavonoids previously isolated from *Citri Reticulatae Pericarpium* provide a very favorable assistance for determination of the characteristics of their precursor ions in positive ion mode. Therefore, the known flavonoids derived from *Citri Reticulatae Pericarpium* were consulted and sorted to build a raw database, which mainly included compound name, molecular formula, as well as structure. Then, the accurate mass of the [M + H]^+^ ion for each compound was calculated by ChemDraw software (version 14.0) to improve the raw database, and besides, the calculated value was saved with four decimal places. The successfully established database was shown in Supplementary Table S1. From Supplementary Table S1, we found that the flavonoids in *Citri Reticulatae Pericarpium* owned the following three characteristics: (1) The molecular weight for each known flavonoid is between 200 and 800, and in order to maximum coverage all of the known and unknown flavonoids in *Citri Reticulatae Pericarpium*, we appropriately expanded the range of their molecular weights as 150–1000. (2) The molecular formulas of flavonoids only contain three elements, namely C, H and O, containing no nitrogen atom, leading to the values of their [M + H]^+^ ions are with odd normal masses. (3) The first number after the decimal point of the value of [M + H]^+^ ion was between zero and three. All of the obtained characteristics of their precursor ions were used to lock the flavonoids from the extracted components.

2.Extracting all compounds in *Citri Reticulatae Pericarpium*

The extract of *Citri Reticulatae Pericarpium* was firstly analyzed in a full MS scan mode, and all compounds in *Citri Reticulatae Pericarpium* were extracted by Compound Discoverer (CD) software (Thermo Scientific, version 3.2.0.421). The extracted workflow was “input files → select spectra → detect compounds → group compounds.” In the “input files” step, the MS full scan data of the extract solution of *Citri Reticulatae Pericarpium* was input in CD software, and in the “select spectra” step, the retention time period (0–30 min) as well as the polarity mode (positive) was set for further processing. A series parameters, such as mass tolerance: 5 ppm, signal-to-noise ratio (S/N) threshold: 3, minimum peak intensity: 100,000, extracted ions: [M + H]^+^ and [M + Na]^+^, minimum element composition: CHO, maximum element composition: C_90_H_190_O_90_Na, etc., were set in the “detect compounds” step to extract all compounds in the input data. The same compound extracted in different addition ways was grouped according to its molecular weight and retention time in the “group compounds” step. All of the extracted compounds were presented by their mass-to-charge ratio (*m/z*), retention time, and peak area.

3.Locking the precursor ions of the flavonoids

The precursor ions meeting the above mentioned characteristics were locked as flavonoids, and then they along with their retention times were constructed in a list. In fact, in this step, the precursor ions of all flavonoids in *Citri Reticulatae Pericarpium* were collected regardless of their intensities, because in the process of extracting compounds, not only higher but also lower intensity ingredients were extracted, in the process of locking precursor ions, the precursor ions of flavonoids were locked based on their characteristics.

4.Targeted MS/MS analysis and structure identification

Each precursor ion in the constructed list was performed targeted MS/MS analysis to obtain fragmentation ions. The flavonoids were identified according to their MS/MS information.

### Flavonoids identification

**Figure 2:**
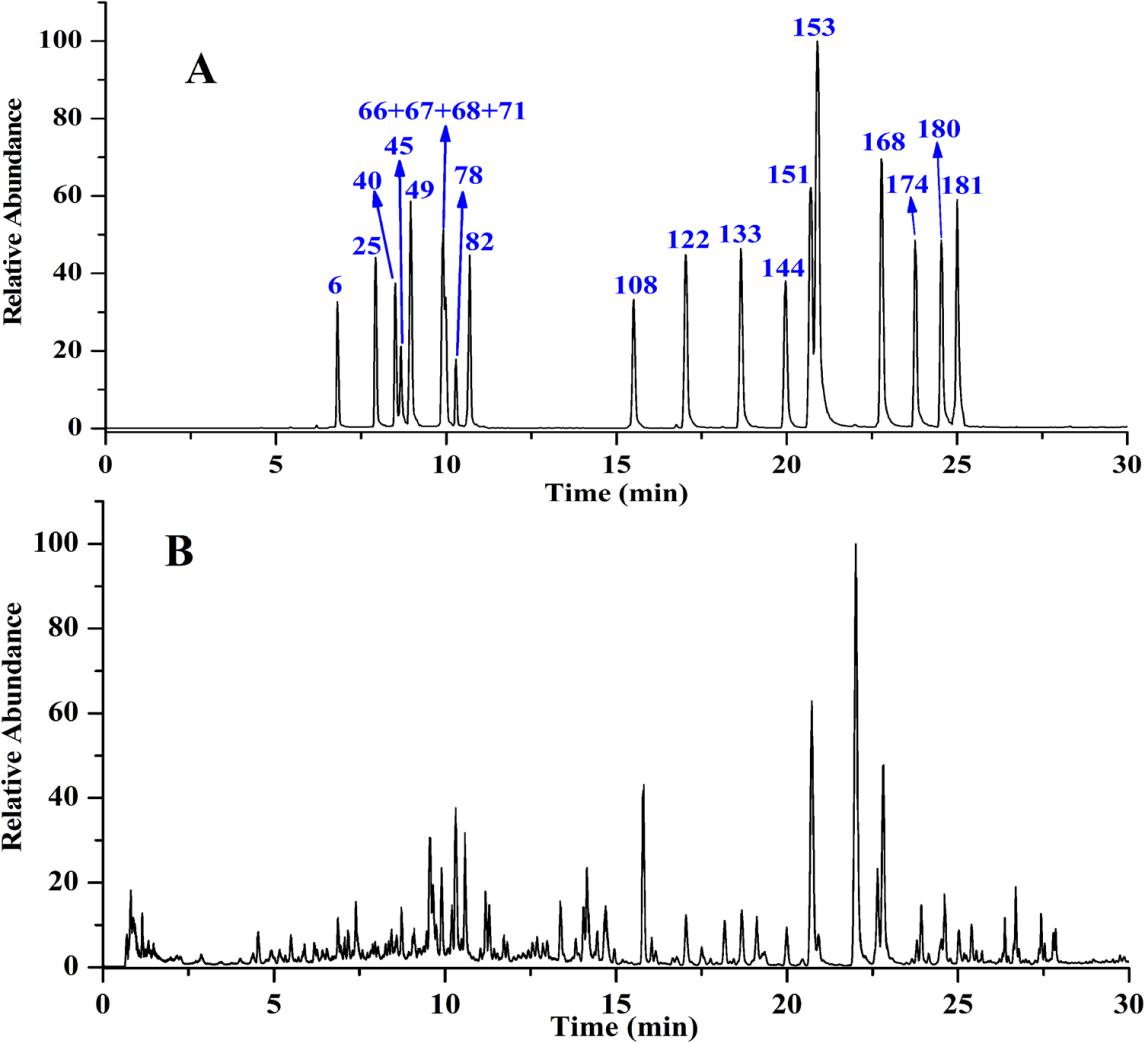
The total ion chromatograms of the reference standards (**A**) and the *Citri Reticulatae Pericarpium* sample (**B**).

In our study, a total of 187 flavonoids, including 54 flavones, 76 flavonols, 51 dihydroflavones and 6 chalcones, were successfully identified (shown in Supplementary Table S2). Their mass deviations are all in the range of − 2.99 to 2.56 ppm, which increases the reliability of the identification results. Among of them, total 21 flavonoids were identified by comparison with their reference standards. The total ion chromatograms of the reference standards and the *Citri Reticulatae Pericarpium* sample were shown in Fig. 2A and B, respectively, and the LC–MS traces of flavonoids were shown in Supplementary Fig. S1.

*For flavones/flavonols characterization* As reported in the literature^17^, flavones/flavonols always yield fragmentation ions by elimination of a series of small molecular units such as CO_2_ (44 Da), H_2_O (18 Da) and CO (28 Da) in their MS/MS spectra. Especially when there is a substituent of OCH_3_ in their structures, the fragmentation ions produced by losses of CH_3_ (15 Da) and OCH_2_ (30 Da) are usually found. For flavonoid glycosides, in their MS/MS spectrum, sugar units are usually firstly lost to form flavonoid aglycones, and then a series of neutral fragments are successively eliminated. These fragmentation pathways were used to deduce the flavones/flavonols in *Citri Reticulatae Pericarpium.* Take the identification of flavonols as an example.

**Figure 3:**
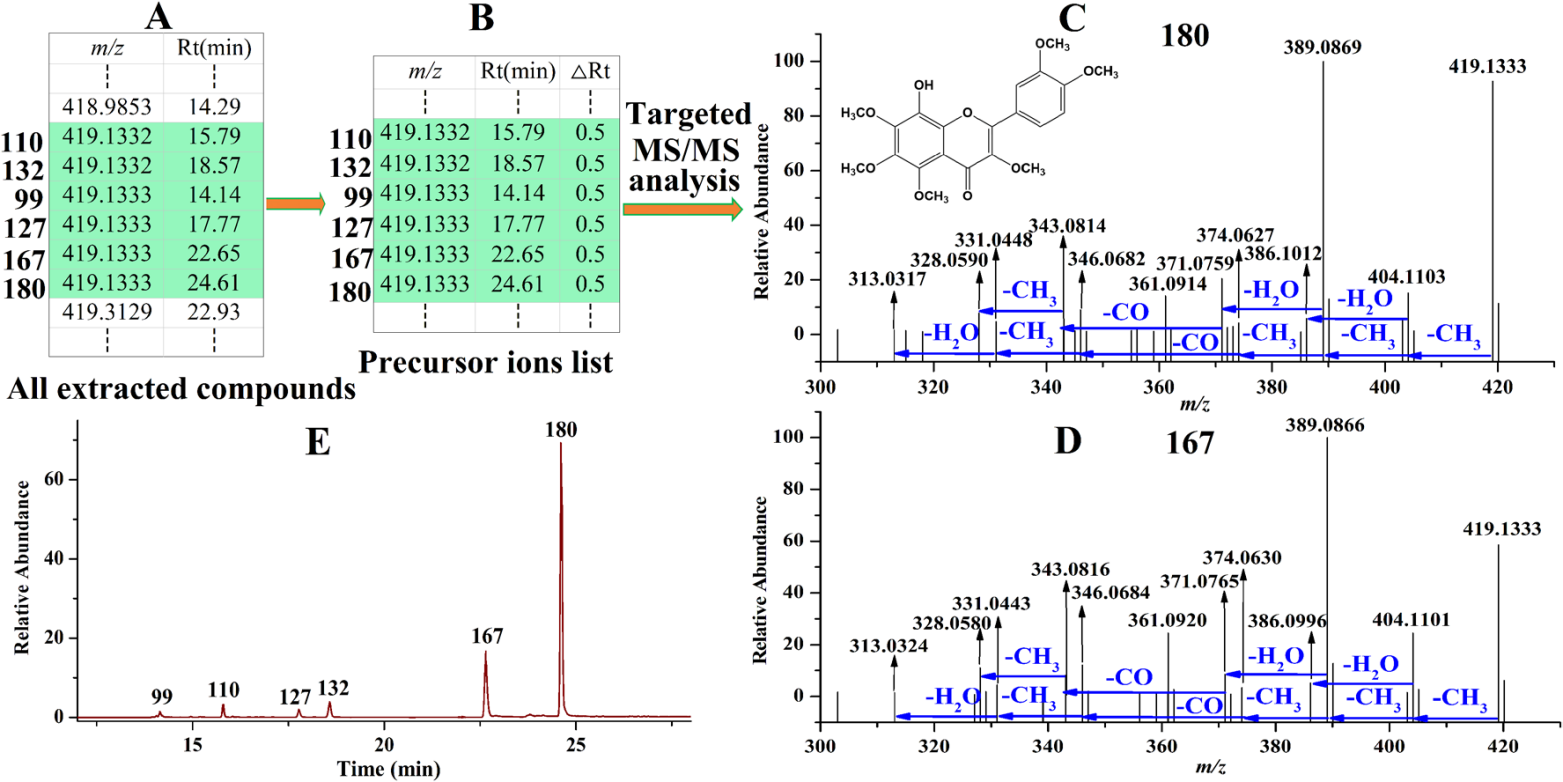
An example for characterization of flavonols. From all the extracted compounds, six compounds were locked as flavonoids (**A**); constructing their precursor ions list (**B**); by performing targeted MS/MS analysis, their MS/MS spectra were obtained such as compound **180** (**C**) and compound **167** (**D**); Extracting the ion chromatograms of the six compounds (**E**).

All compounds in *Citri Reticulatae Pericarpium* were extracted using CD software finally yielding a compound list (shown in Fig. 3A), and among of them, six precursor ions separately extracted at *m/z* 419.1333, 419.1332, 419.1333, 419.1332, 419.1333 and 419.1333 corresponding to compound **99**, **110**, **127**, **132**, **167** and **180**, respectively, were locked as flavonoids. Then, the six precursor ions along with their retention times were constructed in a list (shown in Fig. 3B) for further targeted MS/MS analysis. In the MS/MS spectra of compound **180** (shown in Fig. 3C), the fragmentation ions observed at *m/z* 404.1103, 389.0869, 374.0627, 346.0682, 331.0448 and 313.0317 indicated that CH_3_, CH_3_, CH_3_, CO, CH_3_ and H_2_O were successively eliminated from the precursor ion *m/z* 419.1333, respectively. The ion *m/z* 386.1012 was formed by elimination of H_2_O from the ion *m/z* 404.1103, whereas the ion *m/z* 371.0759 was obtained by loss of H_2_O from the ion *m/z* 389.0869, and then subsequently eliminated CO and CH_3_ to form the ions *m/z* 343.0814 and 328.0590, respectively. By observing the MS/MS spectra of the other five compounds, we found that their fragmentation ions were the same as those of compound **180**, such as compound **167** (shown in Fig. 3D). By comparison with the reference standard, compound **180** was identified as 8-hydroxy-3,5,6,7,3′,4′-hexamethoxyflavone, a known flavone isolated from *Citri Reticulatae Pericarpium*^18^, whereas the other five compounds **99**, **110**, **127**, **132** and **167** were separately identified as its isomer. From Fig. 3C, we found that several OCH_3_ as well as OH substituted on the structure of 8-hydroxy-3,5,6,7,3′,4′-hexamethoxyflavone, and thus, we considered that the difference between compound **180** and its five isomers was the different substitution position of OCH_3_ or OH in flavone structure. Extracting their ion chromatograms, we found that there were indeed six peaks, and there was a big difference in their abundances (shown in Fig. 3E). However, they were all successfully analyzed using our analysis strategy regardless of their intensities. In the same way, the other flavones, flavonols as well as their isomers were successfully identified.

**Figure 4:**
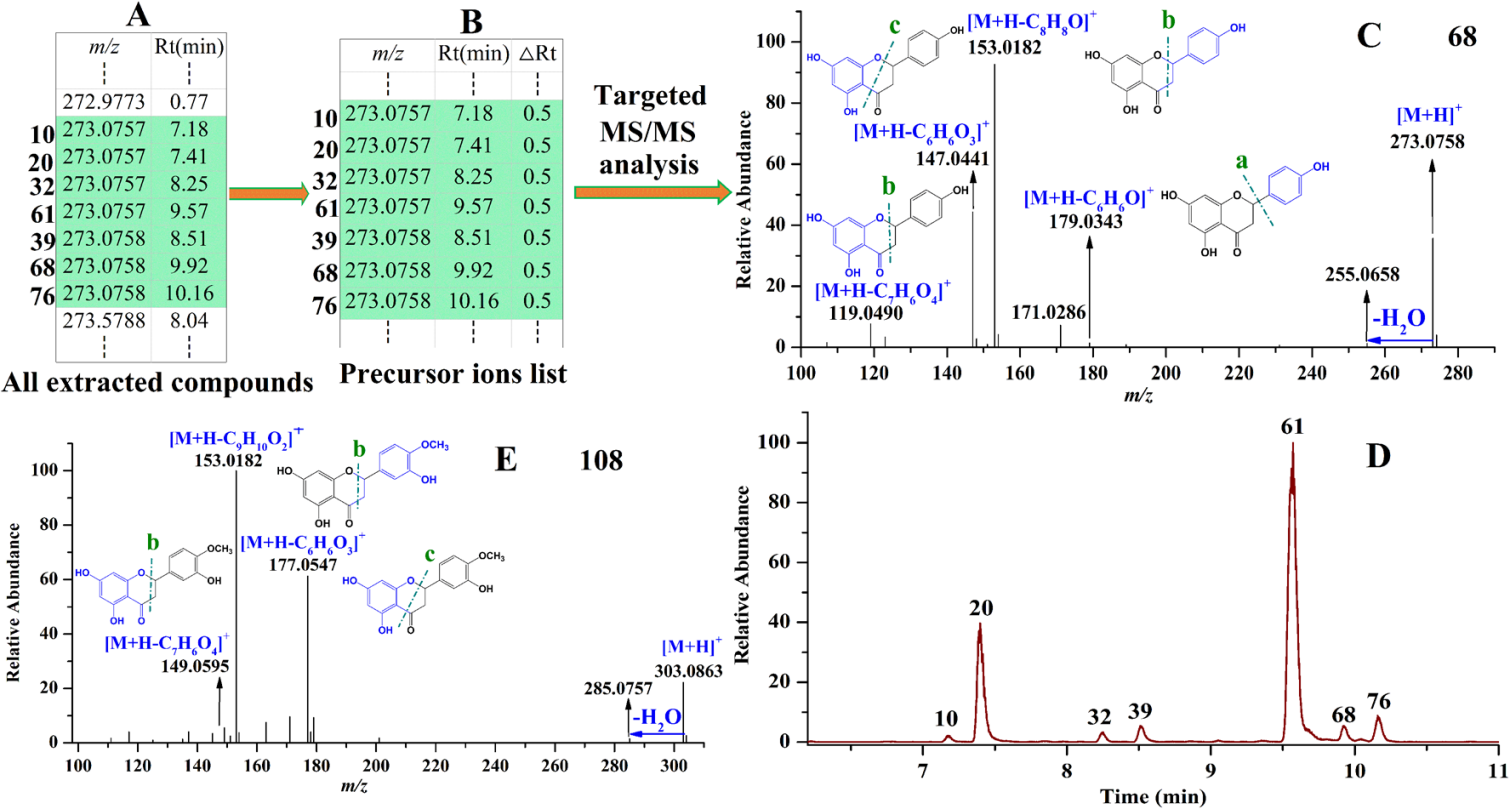
Examples for identification of dihydroflavone. From all the extracted compounds, seven compounds were locked as flavonoids (**A**); constructing their precursor ions list (**B**); by performing targeted MS/MS analysis, their MS/MS spectra was obtained such as compound **68** (**C**); extracting the ion chromatograms of the seven compounds (**D**); the MS/MS spectra of compound **108** (**E**).

*For dihydroflavone characterization* Besides removing some small molecular units like flavones/flavonols, dihydroflavones always produce fragmentation ions by cracking at positions a, b and c in positive ion mode (shown in Fig. 4C)^17^. These fragmentation pattern are beneficial for identifying dihydroflavone compounds in *Citri Reticulatae Pericarpium*. For example, among of all compounds extracted by CD software (shown in Fig. 4A), seven precursor ions separately obtained at *m/z* 273.0757, 273.0757, 273.0757, 273.0757, 273.0758, 273.0758 and 273.0758 corresponding to compound **10**, **20**, **32**, **39**, **61**, **68** and **76**, respectively, were locked as flavonoids, and then their precursor ions were constructed in a list (shown in Fig. 4B) for further targeted MS/MS analysis. From their MS/MS spectra, we found that the seven compounds own the same fragmentation ions. For compound **68** (shown in Fig. 4C), the precursor ion *m/z* 273.0758 produced different fragmentation ions by eliminating different molecular units. For example, the ion *m/z* 179.0343 was formed by loss of C_6_H_6_O (cracking position a), *m/z* 153.0182 was generated by elimination of C_8_H_8_O (cracking position b), *m/z* 147.0441 was obtained by loss of C_6_H_6_O_3_ (cracking position c), and *m/z* 119.0490 was produced by elimination of C_7_H_6_O_4_ (cracking position b). In addition, the fragmentation ion formed by conventionally eliminating H_2_O was also found. Compound **68** was finally identified as naringenin^18^ confirming by its reference standard, whereas the other six compounds were separately identified as its isomer. Extracting their ion chromatograms (shown in Fig. 4D), we found the intensities of the seven components varied greatly, however, they were all successfully characterized. In addition, from Fig. 4C, we found that the abundance of the fragmentation ion *m/z* 179.0343 obtained by cracking at position a was much lower, suggesting the probability of cracking at position a was relatively low, and probably the fragments formed by this cleavage mode would not be found in the MS/MS spectra of some other dihydroflavones. However, the abundances of fragmentation ions *m/z* 153.0182 and *m/z* 147.0441 separately produced by cracking at b and c positions are relatively higher, indicating that dihydroflavones mainly crack at positions b and c, and moreover, we considered cracking at position b has the superiority due to the fragment produced in this cleavage mode owned much higher abundance. These observed fragmentation pattern helped in the identification of other dihydroflavones. For example, in the MS/MS spectra of compound **108** (shown in Fig. 4E), the fragmentation ion *m/z* 285.0757 was formed by elimination of H_2_O from the precursor ion *m/z* 303.0863. When cracking at position b, the precursor ion produced the fragment *m/z* 153.0182 by loss of C_9_H_10_O_2_, whereas when cracking at position c, the precursor ion yielded the ions *m/z* 149.0595 by elimination of C_9_H_6_O_4_ and *m/z* 177.0547 by elimination of C_6_H_6_O_3_. However, the fragments yielded by cracking at position a was not found. Finally, compound **108** was characterized as hesperetin^19^, which was confirmed by comparison with its reference standard.

**Figure 5:**
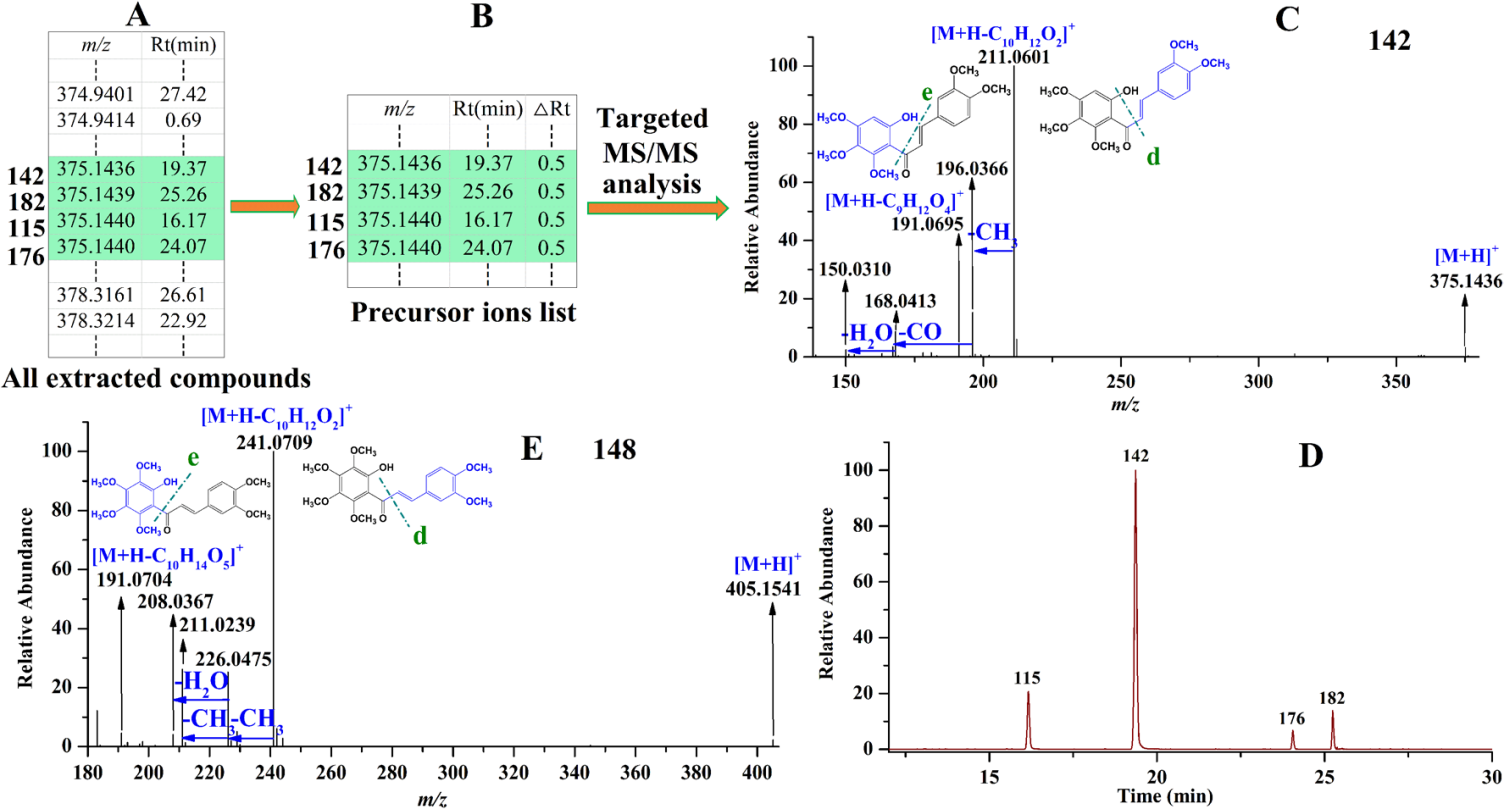
Examples for characterization of chalcones. From all the extracted compounds, four compounds were locked as flavonoids (**A**); constructing their precursor ions list (**B**); by performing targeted MS/MS analysis, their MS/MS spectra were obtained such as compound **142** (**C**); extracting the ion chromatograms of the four compounds (**D**); the MS/MS spectra of compound **148** (**E**).

*For chalcones characterization* Four precursor ions, separately observed at *m/z* 375.1436, 375.1439, 375.1440 and 375.1440 corresponding to compound **115**, **142**, **176** and **182**, respectively, were locked as flavonoids (shown in Fig. 5A). After constructing their precursor ion list (shown in Fig. 5B), they were performed targeted MS/MS analysis to obtain their fragmentation ions. In the MS/MS spectra of compound **142** (shown in Fig. 5C), some fragments produced by removing H_2_O, CH_3_ and CO were found. In addition, the precursor ion *m/z* 375.1436 produced two other important fragments through elimination of different molecular units. One ion observed at *m/z* 211.0601 with a higher abundance was produced by elimination of C_10_H_12_O_2_ (cracking position d), and the other ion obtained at *m/z* 191.0695 with a lower abundance was yielded by loss of C_9_H_12_O_4_ (cracking position e). Compound **142** was ultimately deduced as 2´-hydroxy-3,4,4´,5´,6´-pentamethoxychalcone. The other three compounds **115**, **176** and **182** were separately deduced as its isomer, due to their fragmentation ions were the same as those of compound **142**. Extracting their ion chromatograms (shown in Fig. 5D), we found that the abundance of these four compounds varied significantly, however, they were successfully identified regardless of their intensities. Thus, it can be seen, except for the conventional loss of H_2_O, CH_3_ and CO, chalcones always cleave at position d to produce a higher abundance fragment in MS/MS spectra. However, when cleaving at position e, a weaker abundance fragment is yielded. These fragmentation pathways were used to identify the other chalcones. For example, for compound **148** (shown in Fig. 5E), when the cleavage occurred on position d, the fragment *m/z* 241.0709 with a higher abundance was obtained by elimination of C_10_H_12_O_2_ from the precursor ion *m/z* 405.1541, and when the cleavage occurred on position e, the fragment *m/z* 191.0704 with a lower abundance was yielded by loss of C_10_H_14_O_5_ from the precursor ion *m/z* 405.1541. Finally, compound **148** was tentatively identified as 2´-hydroxy-3,4,3´,4´,5´,6´-hexamethoxychalcone.

**Figure 6:**
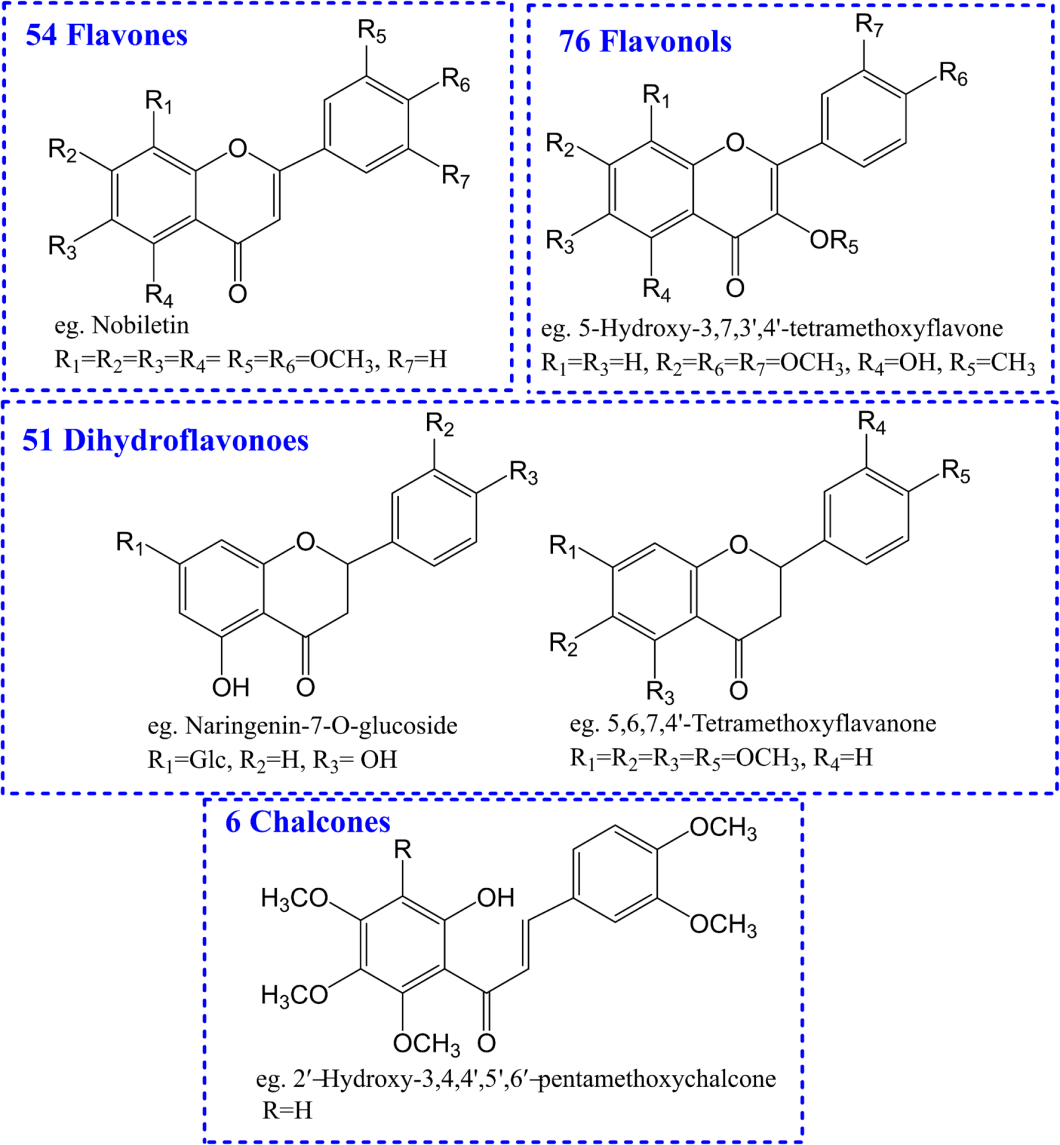
The main structure types of flavonoids identified in *Citri Reticulatae Pericarpium*.

Based on their fragmentation ions, we ultimately characterized all flavonoids in *Citri Reticulatae Pericarpium*. Figure 6 showed the main structural types of flavonoids identified in this study. It was worth noting that, except for flavone, flavonol and dihydroflavone compounds, some chalcones were also characterized in *Citri Reticulatae Pericarpium*, which were reported for the first time in our study. In addition, we found that most of the flavonoids in *Citri Reticulatae Pericarpium* contain multiple OCH_3_, and they are named polymethoxylated flavones. Modern studies have proved that polymethoxylated flavones from natural products have the effects of anti-cancer^20–22^, anti-atherosclerosis^23–25^, anti-inflammatory^26–29^, antioxidant^30^, and treatment of cardiocerebral vascular disease^31,32^, etc. These suggested us that probably the identified polymethoxylated flavones in our study were closely related to the activities of *Citri Reticulatae Pericarpium*.

## Experimental section

### *Citri Reticulatae Pericarpium* sample, reference standards and reagents

*Citri Reticulatae Pericarpium* was supplied by Scientific Research Institute of Beijing Tongrentang Co., Ltd. Total 21 reference standards, including vicenin-2 (**6**), vicenin-3 (**25**), eriocitrin (**40**), isoquercitroside (**45**), troxerutin (**49**), rhoifolin (**66**), naringin (**67**), naringenin (**68**), naringenin-7-O-glucoside (**71**), hesperidin (**78**), neohesperidin (**82**), hesperetin (**108**), isosinensetin (**122**), sinensetin (**133**), quercetagetin-3,5,6,7,3′,4′-hexamethyl ether (**144**), nobiletin (**151**), 4´,5,6,7-tetramethoxyflavone (**153**), tangeretin (**168**), 5-demethylnobiletin (**174**), 8-hydroxy-3,5,6,7,3′,4′-hexamethoxyflavone (**180**), 5-hydroxy-3,7,3',4'-tetramethoxyflavone (**181**), were purchased from Shanghai Yuanye Bio-Technology Co., Ltd. (Shanghai, China). The purity of all the reference standards was > 98%. The distilled water was obtained from Watsons, whereas LC–MS grade formic acid was obtained from Fisher-Scientific (Fair Lawn, USA). The LC–MS-grade acetonitrile and methanol were purchased from Merck (Germany).

### Sample and standard solution preparations

The *Citri Reticulatae Pericarpium* was pulverized into powder (the dimension of the obtained powder just like flour), and then 1.0 g of the power was extracted ultrasonically for 30 min with 10 mL 70% methanol at 25 °C. The extracted solution was then filtered through a 0.22 μm nylon filter membrane before analysis. Each reference standard (1.0 mg) was separately dissolved in 1 mL methanol to obtain its stock solution (1.0 mg/mL), and then an appropriate amount of each stock solution was mixed to obtain the standard stock solution storing at 4 °C until analysis. The 21 reference standards were used only for the characterization confirmation.

### Chromatographic and mass spectrometric conditions

A Vanquish™ Flex UPLC system (Thermo Scientific, USA) equipped with a binary pump and a thermostatted column compartment was used to analyze the extract of *Citri Reticulatae Pericarpium*. Complicate components were successfully separated on a Waters ACQUITY UPLC® BEH C_18_ column (2.1 × 100 mm, 1.7 μm) coupled with a ACQUITY UPLC® BEH C_18_ VanGuard™ Pre-Column (2.1 × 5 mm, 1.7 μm) using mobile phase A (0.1% formic acid/water, v/v) and mobile phase B (acetonitrile) by the following gradient elution program: 0–7 min, 2–20% B; 7–10 min, 20–25% B; 10–20 min, 25–40% B; 20–25 min, 40–65% B; 25–30 min, 65–95% B. The temperature of column chamber was set at 35 °C whereas the temperature of sampler module was set at 8 °C. The injection volume was 2 μL and the flow rate was 0.3 mL/min.

An Orbitrap Exploris 240 mass spectrometer (Thermo Scientific, USA) equipped with a Heated ESI source was used to acquire the mass spectra. Due to the [M + H]^+^ ions of the flavonoids exhibit high responses in positive ion mode, the instrument was operated in positive ion mode. The optimized HR MS parameters were described as follows: ion spray voltage: 3400 V, sheath gas: 5.08 L/min, auxiliary gas: 9.37 L/min, ion transfer tube temperature: 320 °C, vaporizer temperature: 350 °C, scan range (*m/z*): 150–1000, collision energy mode: stepped, collision energy type: normalized, HCD collision energy (%): 30, 45, 60. The full scan was operated at a mass resolution of 60,000, whereas the targeted MS/MS analysis was operated at a mass resolution of 15,000. Internal calibration source Thermo Scientific EASY-IC™ was adopted to calibrate the entire mass range.

### Data acquisition and data processing

Data acquisition was performed on Thermo Xcalibur software (version 4.5), and the extracted solution of *Citri Reticulatae Pericarpium* was first analyzed in a full MS scan mode so as to minimize the losses of signals. Data processing were performed on Free Style™ 1.8 SP1 software and Compound Discoverer™ software (Thermo Scientific™, version 3.2.0.421). The flavonoids were identified according to the obtained MS/MS information.

## Conclusions

High resolution mass spectrometry (HR MS) plays an important role in the qualitative analysis of secondary metabolites, and always the auto-MS/MS mode, in which only about three most intense ions are selected, is used. However, it encounters a drawback in chemical substances identification when samples contain many overlapping signals. In our experiment, an analysis strategy based on precursor ions locked and targeted MS/MS analysis was proposed to comprehensively clarify the secondary metabolites-flavonoids in *Citri Reticulatae Pericarpium*, and as a result, total 187 flavonoids were successfully identified. The number of identified flavonoids far exceeded those currently isolated from *Citri Reticulatae Pericarpium* and previously identified by high-resolution mass spectrometry. The greatest advantage of our analytical strategy was that, the precursor ions of the flavonoids were locked according to the characteristics of their precursor ions rather than their intensities, which meant the flavonoids with not only high but also low intensities could be identified.

In addition, it was the first time to systematically clarify the flavonoids in *Citri Reticulatae Pericarpium*, our result will expand the current knowledge on the chemistry of the investigated drug. Our established strategy can provide a reference for the analysis of flavonoids in other traditional Chinese medicines.

### Supplementary Information

Supplementary Information.

## Data Availability

All data generated or analyzed during this study are included in this published article (and its Supplementary Information files).
