# Supplementary material for: Comprehensive identifying flavonoids in Citri Reticulatae Pericarpium using a novel strategy based on precursor ions locked and targeted MS/MS analysis
Source: Sci Rep. 2024 Apr 27;14:9679. doi: 10.1038/s41598-024-60415-w (PMC11055944; doi:10.1038/s41598-024-60415-w)
Supplement: Supplementary file 1 — Supplementary Information. [file 41598_2024_60415_MOESM1_ESM.doc]

##### Comprehensive characterizing flavonoids in *Citri Reticulatae Pericarpium* using a novel strategy based on precursor ions locked and targeted MS/MS analysis

Hong-Ping Wang ^1^, Zhao-Zhou Lin ^2^, Hui Wang ^1^,, Xuan Yang ^1,*^ , Nan Niu ^1,*^

^1^ Scientific Research Institute of Beijing Tongrentang Co., Ltd., Beijing 100011, China

^2^ Beijing Zhongyan Tongrentang Pharmaceutical R & D Co., Ltd., National Engineering Research Center for R&D of TCM Multi-ingredient Drugs, Beijing 100079, China

 Corresponding author. Tel.: +86 10 87632655; fax: +86 10 87632655. *E-mail address*: yx200812@163.com

**Supplementary Information**

Table S1 The flavonoids isolated from *Citri Reticulatae Pericarpium*.

| No. | Compound Name | Molecular Formulae | The Calculated value of [M+H]^+^ | Structure |
| --- | --- | --- | --- | --- |
| 1 | [Nobiletin](https://www.chemsrc.com/cas/478-01-3_122104.html) | C_21_H_22_O_8_ | 403.1393 |  |
| 2 | Dihydroxy-trimethoxyflavone | C_18_H_16_O_7_ | 345.0974 |  |
| 3 | [Tangeretin](https://www.chemsrc.com/cas/481-53-8_950947.html) | C_20_H_20_O_7_ | 373.1287 |  |
| 4 | 3,3′,4′,5,6,7,8-Heptamethoxyflavone | C_22_H_24_O_9_ | 433.1499 |  |
| 5 | Quercetagetin-3,7,3′,4′-tetramethyl ether | C_19_H_18_O_8_ | 375.1080 |  |
| 6 | [Isosinensetin](https://www.chemsrc.com/cas/17290-70-9_964698.html) | C_20_H_20_O_7_ | 373.1287 |  |
| 7 | [5-Demethylnobiletin](https://www.chemsrc.com/cas/2174-59-6_964689.html) | C_20_H_20_O_8_ | 389.1236 |  |
| 8 | Vicenin-3 | C_26_H_28_O_14_ | 565.1557 |  |
| 9 | [Kaempferol-3-O-rutinoside](https://www.chemsrc.com/cas/17297-56-2_89274.html) | C_27_H_30_O_15_ | 595.1663 |  |
| 10 | [Rhoifolin](https://www.chemsrc.com/cas/17306-46-6_141870.html) | C_27_H_30_O_14_ | 579.1714 |  |
| 11 | Quercetagetin-3,5,6,7,3′,4′-hexamethyl ether | C_21_H_22_O_8_ | 403.1393 |  |
| 12 | [Sinensetin](https://www.chemsrc.com/cas/2306-27-6_141840.html) | [C](https://pubchem.ncbi.nlm.nih.gov/#query=C20H20O7)_20_H_20_O_7_ | 373.1287 |  |
| 13 | 8-Hydroxy-3,5,6,7,3′,4′-hexamethoxyflavone | C_21_H_22_O_9_ | 419.1342 |  |
| 14 | 5,4´-Dihydroxy-3,6,7,8,3´-pentamethoxyflavone | C_20_H_20_O_9_ | 405.1186 |  |
| 15 | 5-Hydroxy-3,7,3',4'-tetramethoxyflavone | C_19_H_18_O_7_ | 359.1131 |  |
| 16 | 4´,5,6,7-Tetramethoxyflavone | C_19_H_18_O_6_ | 343.1182 |  |
| 17 | [Isoquercitroside](https://www.chemsrc.com/cas/21637-25-2_599763.html) | C_21_H_20_O_12_ | 465.1033 |  |
| 18 | [Eriocitrin](https://www.chemsrc.com/cas/13463-28-0_122092.html) | C_27_H_32_O_15_ | 597.1819 |  |
| 19 | [Hesperidin](https://www.chemsrc.com/cas/520-26-3_402114.html) | C_28_H_34_O_15_ | 611.1976 |  |
| 20 | [Naringin](https://www.chemsrc.com/cas/10236-47-2_402113.html) | C_27_H_32_O_14_ | 581.1870 |  |
| 21 | Naringenin-7-O-glucoside | C_21_H_22_O_10_ | 435.1291 |  |
| 22 | [Neohesperidin](https://www.chemsrc.com/cas/13241-33-3_402164.html) | C_28_H_34_O_15_ | 611.1976 |  |
| 23 | [Hesperetin](https://www.chemsrc.com/cas/520-33-2_894795.html) | C_16_H_14_O_6_ | 303.0869 |  |
| 24 | Naringenin | C_15_H_12_O_5_ | 273.0763 |  |
| 25 | [5,6,7,4'-Tetramethoxyflavanone](https://www.chemsrc.com/cas/72943-90-9_1680019.html) | C_19_H_20_O_6_ | 345.1338 |  |

Table S2 The flavonoids characterized from *Citri Reticulatae Pericarpium*.

| No. | *t*_R_ (min) | Molecular formulae | Measured value (*m/z*) | Diff (ppm) | Product ions | Compound name | Subclasses |
| --- | --- | --- | --- | --- | --- | --- | --- |
| 1 | 5.87 | C_27_H_30_O_15_ | 595.1659 | -0.67 | 541.1353[M+H-3H_2_O]^+^,  523.1260[M+H-4H_2_O]^+^,  433.1002[M+H-Glc]^+^,  271.0600[M+H-2Glc]^+^ | Vicenin-2 isomer | Flavone |
| 2 | 5.90 | C_21_H_20_O_12_ | 465.1028 | -1.08 | 303.0500[M+H-Glc]^+^,  285.0385[M+H-Glc-H_2_O]^+^,  257.0445[M+H-Glc-H_2_O-CO]^+^,  229.0499[M+H-Glc-H_2_O-2CO]^+^,  201.0555[M+H-Glc-H_2_O-3CO]^+^ | [Isoquercitroside](https://www.chemsrc.com/cas/21637-25-2_599763.html) isomer | Flavonol |
| 3 | 6.46 | C_15_H_10_O_5_ | 271.0600 | -2.21 | 243.0641[M+H-CO]^+^,  215.0699[M+H-2CO]^+^ | [Apigenin](https://www.chemsrc.com/cas/520-36-5_254337.html) isomer | Flavone |
| 4 | 6.47 | C_27_H_32_O_15_ | 597.1814 | -0.84 | 289.0708[M+H-Rha-Glc]^+^ | [Eriocitrin](https://www.chemsrc.com/cas/13463-28-0_122092.html) isomer | Dihydroflavonoid |
| 5 | 6.75 | C_16_H_12_O_6_ | 301.0706 | -1.99 | 286.0463[M+H-CH_3_]^+^ | Tectorigenin isomer | Flavone |
| 6 | 6.87 | C_27_H_30_O_15_ | 595.1651 | -2.02 | 541.1342[M+H-3H_2_O]^+^,  523.1208[M+H-4H_2_O]^+^,  433.0931[M+H-Glc]^+^,  271.0602[M+H-2Glc]^+^ | Vicenin-2 *^a^* [1] | Flavone |
| 7 | 6.88 | C_26_H_28_O_14_ | 565.1553 | -0.71 | 433.1112[M+H-Ara]^+^,  415.1034[M+H-Ara-H_2_O]^+^,  397.0914[M+H-Ara-2H_2_O]^+^,  379.0835[M+H-Ara-3H_2_O]^+^,  367.0822[M+H-Ara-2H_2_O-OCH_2_]^+^ | Vicenin-3 isomer | Flavone |
| 8 | 6.94 | C_15_H_10_O_5_ | 271.0601 | -1.84 | 243.0650[M+H-CO]^+^,  215.0703[M+H-2CO]^+^ | [Apigenin](https://www.chemsrc.com/cas/520-36-5_254337.html) isomer | Flavone |
| 9 | 7.18 | C_16_H_12_O_6_ | 301.0707 | -1.66 | 286.0488[M+H-CH_3_]^+^,  273.0762[M+H-CO]^+^ | Tectorigenin [1] | Flavone |
| 10 | 7.18 | C_15_H_12_O_5_ | 273.0757 | -2.20 | 153.0177[M+H-C_8_H_8_O]^+^,  147.0444[M+H-C_6_H_6_O_3_]^+^,  119.0492[M+H-C_7_H_6_O_4_]^+^ | Naringenin isomer | Dihydroflavonoid |
| 11 | 7.27 | C_15_H_10_O_5_ | 271.0601 | -1.84 | 215.0698[M+H-2CO]^+^ | [Apigenin](https://www.chemsrc.com/cas/520-36-5_254337.html) [2] | Flavone |
| 12 | 7.29 | C_16_H_12_O_6_ | 301.0704 | -2.66 | 286.0429[M+H-CH_3_]^+^ | Tectorigenin isomer | Flavone |
| 13 | 7.29 | C_27_H_30_O_15_ | 595.1660 | -0.50 | 541.1332[M+H-3H_2_O]^+^,  523.1232[M+H-4H_2_O]^+^,  433.1104[M+H-Glc]^+^,  271.0604[M+H-2Glc]^+^ | Vicenin-2 isomer | Flavone |
| 14 | 7.31 | C_27_H_32_O_15_ | 597.1814 | -0.84 | 289.0705[M+H-Rha-Glc]^+^ | [Eriocitrin](https://www.chemsrc.com/cas/13463-28-0_122092.html) isomer | Dihydroflavonoid |
| 15 | 7.31 | C_21_H_22_O_10_ | 435.1285 | -1.38 | 273.0764[M+H-Glc]^+^ | Naringenin-7-O-glucoside isomer | Dihydroflavonoid |
| 16 | 7.36 | C_27_H_30_O_15_ | 595.1663 | 0.00 | 271.0602[M+H-2Glc]^+^ | Vicenin-2 isomer | Flavone |
| 17 | 7.39 | C_27_H_32_O_15_ | 597.1813 | -1.00 | 289.0709[M+H-Rha-Glc]^+^ | [Eriocitrin](https://www.chemsrc.com/cas/13463-28-0_122092.html) isomer | Dihydroflavonoid |
| 18 | 7.40 | C_27_H_32_O_14_ | 581.1862 | -1.38 | 273.0757[M+H-Rha-Glc]^+^ | [Naringin](https://www.chemsrc.com/cas/10236-47-2_402113.html) isomer | Dihydroflavonoid |
| 19 | 7.40 | C_21_H_22_O_10_ | 435.1284 | -1.61 | 273.0755[M+H-Glc]^+^ | Naringenin-7-O-glucoside isomer | Dihydroflavonoid |
| 20 | 7.41 | C_15_H_12_O_5_ | 273.0757 | -2.20 | 179.0341[M+H-C_6_H_6_O]^+^,  153.0183[M+H-C_8_H_8_O]^+^,  147.0441[M+H-C_6_H_6_O_3_]^+^,  119.0491[M+H-C_7_H_6_O_4_]^+^ | Naringenin isomer | Dihydroflavonoid |
| 21 | 7.57 | C_21_H_20_O_12_ | 465.1030 | -0.65 | 303.0501[M+H-Glc]^+^ | [Isoquercitroside](https://www.chemsrc.com/cas/21637-25-2_599763.html) isomer | Flavonol |
| 22 | 7.67 | C_27_H_30_O_15_ | 595.1661 | -0.34 | 541.1374[M+H-3H_2_O]^+^,  523.1251[M+H-4H_2_O]^+^,  433.1046[M+H-Glc]^+^,  271.0604[M+H-2Glc]^+^ | Vicenin-2 isomer | Flavone |
| 23 | 7.81 | C_27_H_30_O_15_ | 595.1661 | -0.34 | 541.1332[M+H-3H_2_O]^+^,  523.1213[M+H-4H_2_O]^+^,  433.0940[M+H-Glc]^+^ | Vicenin-2 isomer | Flavone |
| 24 | 7.84 | C_16_H_12_O_6_ | 301.0706 | -1.99 | 286.0476[M+H-CH_3_]^+^ | Tectorigenin isomer | Flavone |
| 25 | 7.94 | C_26_H_28_O_14_ | 565.1553 | -0.71 | 433.0924[M+H-Ara]^+^,  415.0860[M+H-Ara-H_2_O]^+^,  397.0920[M+H-Ara-2H_2_O]^+^,  379.0815[M+H-Ara-3H_2_O]^+^,  367.0800[M+H-Ara-2H_2_O-OCH_2_]^+^ | Vicenin-3 *^a^* [3] | Flavone |
| 26 | 8.05 | C_21_H_20_O_12_ | 465.1027 | -1.29 | 303.0502[M+H-Glc]^+^,  285.0407[M+H-Glc-H_2_O]^+^,  257.0421[M+H-Glc-H_2_O-CO]^+^ | [Isoquercitroside](https://www.chemsrc.com/cas/21637-25-2_599763.html) isomer | Flavonol |
| 27 | 8.05 | C_27_H_30_O_15_ | 595.1661 | -0.34 | 433.1127[M+H-Glc]^+^,  271.0590[M+H-2Glc]^+^ | Vicenin-2 isomer | Flavone |
| 28 | 8.11 | C_27_H_30_O_15_ | 595.1661 | -0.34 | 433.1131[M+H-Glc]^+^,  271.0604[M+H-2Glc]^+^ | Vicenin-2 isomer | Flavone |
| 29 | 8.14 | C_27_H_30_O_14_ | 579.1708 | -1.04 | 433.1099[M+H-Rha]^+^,  415.1030[M+H-Rha-H_2_O]^+^ | [Rhoifolin](https://www.chemsrc.com/cas/17306-46-6_141870.html) isomer | Flavone |
| 30 | 8.24 | C_21_H_22_O_10_ | 435.1290 | -0.23 | 273.0766[M+H-Glc]^+^ | Naringenin-7-O-glucoside isomer | Dihydroflavonoid |
| 31 | 8.25 | C_26_H_28_O_14_ | 565.1547 | -1.77 | 433.1145[M+H-Ara]^+^,  415.1015[M+H-Ara-H_2_O]^+^,  397.0918[M+H-Ara-2H_2_O]^+^,  379.0815[M+H-Ara-3H_2_O]^+^,  367.0815[M+H-Ara-2H_2_O-OCH_2_]^+^,  361.0714[M+H-Ara-4H_2_O]^+^,  349.0718[M+H-Ara-3H_2_O-OCH_2_]^+^,  271.0605[M+H-Ara-Glc]^+^ | Vicenin-3 isomer | Flavone |
| 32 | 8.25 | C_15_H_12_O_5_ | 273.0757 | -2.20 | 179.0339[M+H-C_6_H_6_O]^+^,  153.0182[M+H-C_8_H_8_O]^+^,  147.0441[M+H-C_6_H_6_O_3_]^+^,  119.0493[M+H-C_7_H_6_O_4_]^+^ | Naringenin isomer | Dihydroflavonoid |
| 33 | 8.26 | C_28_H_34_O_15_ | 611.1970 | -0.98 | 303.0865[M+H-Rha-Glc]^+^ | [Hesperidin](https://www.chemsrc.com/cas/520-26-3_402114.html) isomer/[Neohesperidin](https://www.chemsrc.com/cas/13241-33-3_402164.html) isomer | Dihydroflavonoid |
| 34 | 8.26 | C_16_H_14_O_6_ | 303.0862 | -2.31 | 285.0746[M+H-H_2_O]^+^,  177.0547[M+H-C_6_H_6_O_3_]^+^,  153.0181[M+H-C_9_H_10_O_2_]^+^,  149.0595[M+H-C_9_H_6_O_4_]^+^ | [Hesperetin](https://www.chemsrc.com/cas/520-33-2_894795.html) isomer | Dihydroflavonoid |
| 35 | 8.39 | C_16_H_14_O_6_ | 303.0863 | -1.98 | 285.0777[M+H-H_2_O]^+^,  177.0545[M+H-C_6_H_6_O_3_]^+^,  153.0183[M+H-C_9_H_10_O]^+^,  149.0596[M+H-C_9_H_6_O_4_]^+^ | [Hesperetin](https://www.chemsrc.com/cas/520-33-2_894795.html) isomer | Dihydroflavonoid |
| 36 | 8.41 | C_26_H_28_O_14_ | 565.1550 | -1.24 | 433.1134[M+H-Ara]^+^,  415.1025[M+H-Ara-H_2_O]^+^,  397.0922[M+H-Ara-2H_2_O]^+^,  379.0814[M+H-Ara-3H_2_O]^+^,  367.0821[M+H-Ara-2H_2_O-OCH_2_]^+^,  271.0610[M+H-Ara-Glc]^+^ | Vicenin-3 isomer | Flavone |
| 37 | 8.41 | C_27_H_30_O_14_ | 579.1708 | -1.04 | 433.1131[M+H-Rha]^+^,  415.1030[M+H-Rha-H_2_O]^+^,  397.0916[M+H-Rha-2H_2_O]^+^,  379.0810[M+H-Rha-3H_2_O]^+^,  271.0613[M+H-Rha-Glc]^+^ | [Rhoifolin](https://www.chemsrc.com/cas/17306-46-6_141870.html) isomer | Flavone |
| 38 | 8.43 | C_21_H_20_O_12_ | 465.1028 | -1.08 | 303.0500[M+H-Glc]^+^,  285.0395[M+H-Glc-H_2_O]^+^,  257.0437[M+H-Glc-H_2_O-CO]^+^,  229.0497[M+H-Glc-H_2_O-2CO]^+^,  201.0542[M+H-Glc-H_2_O-3CO]^+^ | [Isoquercitroside](https://www.chemsrc.com/cas/21637-25-2_599763.html) isomer | Flavonol |
| 39 | 8.51 | C_15_H_12_O_5_ | 273.0758 | -1.83 | 179.0341[M+H-C_6_H_6_O]^+^,  153.0183[M+H-C_8_H_8_O]^+^,  147.0441[M+H-C_6_H_6_O_3_]^+^,  119.0492[M+H-C_7_H_6_O_4_]^+^ | Naringenin isomer | Dihydroflavonoid |
| 40 | 8.52 | C_27_H_32_O_15_ | 597.1811 | -1.34 | 289.0706[M+H-Rha-Glc]^+^ | [Eriocitrin](https://www.chemsrc.com/cas/13463-28-0_122092.html) *^a^* [4] | Dihydroflavonoid |
| 41 | 8.57 | C_21_H_20_O_12_ | 465.1026 | -1.51 | 303.0497[M+H-Glc]^+^ | [Isoquercitroside](https://www.chemsrc.com/cas/21637-25-2_599763.html) isomer | Flavonol |
| 42 | 8.58 | C_27_H_30_O_14_ | 579.1708 | -1.04 | 415.1093[M+H-Rha-H_2_O]^+^,  397.0886[M+H-Rha-2H_2_O]^+^,  379.0814[M+H-Rha-3H_2_O]^+^,  271.0591[M+H-Rha-Glc]^+^ | [Rhoifolin](https://www.chemsrc.com/cas/17306-46-6_141870.html) isomer | Flavone |
| 43 | 8.60 | C_27_H_30_O_15_ | 595.1656 | -1.18 | 449.1085[M+H-Rha]^+^,  427.1028[M+H-Rha-H_2_O]^+^,  409.0909[M+H-Rha-2H_2_O]^+^,  397.0925[M+H-Rha-H_2_O-OCH_2_]^+^,  391.0803[M+H-Rha-3H_2_O]^+^,  379.0825[M+H-Rha-2H_2_O-OCH_2_]^+^,  287.0551[M+H-Rha-Glc]^+^ | [Kaempferol-3-O-rutinoside](https://www.chemsrc.com/cas/17297-56-2_89274.html) [5] | Flavonol |
| 44 | 8.65 | C_27_H_30_O_15_ | 595.1656 | -1.18 | 449.1080[M+H-Rha]^+^,  427.1038[M+H-Rha-H_2_O]^+^,  409.0906[M+H-Rha-2H_2_O]^+^,  287.0552[M+H-Rha-Glc]^+^ | [Kaempferol-3-O-rutinoside](https://www.chemsrc.com/cas/17297-56-2_89274.html) isomer | Flavonol |
| 45 | 8.72 | C_21_H_20_O_12_ | 465.1029 | -0.86 | 303.0500[M+H-Glc]^+^,  285.0394[M+H-Glc-H_2_O]^+^,  257.0445[M+H-Glc-H_2_O-CO]^+^,  229.0499[M+H-Glc-H_2_O-2CO]^+^,  201.0557[M+H-Glc-H_2_O-3CO]^+^ | [Isoquercitroside](https://www.chemsrc.com/cas/21637-25-2_599763.html) *^a^* [6] | Flavonol |
| 46 | 8.82 | C_27_H_30_O_15_ | 595.1657 | -1.01 | 427.1026[M+H-Rha-H_2_O]^+^,  409.0913[M+H-Rha-2H_2_O]^+^,  397.0914[M+H-Rha-H_2_O-OCH_2_]^+^,  391.0809[M+H-Rha-3H_2_O]^+^,  379.0800[M+H-Rha-2H_2_O-OCH_2_]^+^,  287.0553[M+H-Rha-Glc]^+^ | [Kaempferol-3-O-rutinoside](https://www.chemsrc.com/cas/17297-56-2_89274.html) isomer | Flavonol |
| 47 | 8.94 | C_27_H_30_O_15_ | 595.1660 | -0.50 | 427.1032M+H-Rha-H_2_O]^+^,  409.0928[M+H-Rha-2H_2_O]^+^,  397.0907[M+H-Rha-H_2_O-OCH_2_]^+^,  287.0561[M+H-Rha-Glc]^+^ | [Kaempferol-3-O-rutinoside](https://www.chemsrc.com/cas/17297-56-2_89274.html) isomer | Flavonol |
| 48 | 8.94 | C_27_H_32_O_14_ | 581.1864 | -1.03 | 273.0767[M+H-Rha-Glc]^+^ | [Naringin](https://www.chemsrc.com/cas/10236-47-2_402113.html) isomer | Dihydroflavonoid |
| 49 | 8.95 | C_33_H_42_O_19_ | 743.2392 | -0.94 | 435.1284[M+H-Rha-Glc]^+^,  391.1019[M+H-Rha-Glc-CO_2_]^+^,  373.0927[M+H-Rha-Glc-CO_2_-H_2_O]^+^ | [Troxerutin](https://www.chemsrc.com/cas/7085-55-4_672587.html) *^a^* [7] | Flavonol |
| 50 | 8.97 | C_27_H_30_O_14_ | 579.1709 | -0.86 | 433.1132[M+H-Rha]^+^,  271.0613[M+H-Rha-Glc]^+^ | [Rhoifolin](https://www.chemsrc.com/cas/17306-46-6_141870.html) isomer | Flavone |
| 51 | 9.01 | C_27_H_30_O_15_ | 595.1658 | -0.84 | 427.1028[M+H-Rha-H_2_O]^+^,  409.0925[M+H-Rha-2H_2_O]^+^,  397.0906[M+H-Rha-H_2_O-OCH_2_]^+^,  287.0528[M+H-Rha-Glc]^+^ | [Kaempferol-3-O-rutinoside](https://www.chemsrc.com/cas/17297-56-2_89274.html) isomer | Flavonol |
| 52 | 9.05 | C_27_H_30_O_14_ | 579.1709 | -0.86 | 271.0601[M+H-Rha-Glc]^+^ | [Rhoifolin](https://www.chemsrc.com/cas/17306-46-6_141870.html) isomer | Flavone |
| 53 | 9.09 | C_16_H_14_O_6_ | 303.0863 | -1.98 | 285.0752[M+H-H_2_O]^+^,  177.0549[M+H-C_6_H_6_O_3_]^+^,  153.0182[M+H-C_9_H_10_O]^+^,  149.0597[M+H-C_9_H_6_O_4_]^+^ | [Hesperetin](https://www.chemsrc.com/cas/520-33-2_894795.html) isomer | Dihydroflavonoid |
| 54 | 9.24 | C_26_H_28_O_14_ | 565.1552 | -0.88 | 415.1011[M+H-Ara-H_2_O]^+^,  397.0971[M+H-Ara-2H_2_O]^+^,  379.0846[M+H-Ara-3H_2_O]^+^,  367.0822[M+H-Ara-2H_2_O-OCH_2_]^+^,  349.0722[M+H-Ara-3H_2_O-OCH_2_]^+^,  271.0595[M+H-Ara-Glc]^+^ | Vicenin-3 isomer | Flavone |
| 55 | 9.27 | C_27_H_30_O_15_ | 595.1658 | -0.84 | 449.1115[M+H-Rha]^+^,  287.0552[M+H-Rha-Glc]^+^ | [Kaempferol-3-O-rutinoside](https://www.chemsrc.com/cas/17297-56-2_89274.html) isomer | Flavonol |
| 56 | 9.40 | C_16_H_12_O_6_ | 301.0703 | -2.99 | 286.0473[M+H-CH_3_]^+^,  258.0527[M+H-CH_3_-CO]^+^ | Tectorigenin isomer | Flavone |
| 57 | 9.51 | C_16_H_14_O_6_ | 303.0863 | -1.98 | 285.0785[M+H-H_2_O]^+^,  177.0546[M+H-C_6_H_6_O_3_]^+^,  153.0181[M+H-C_9_H_10_O_2_]^+^,  149.0593[M+H-C_9_H_6_O_4_]^+^ | [Hesperetin](https://www.chemsrc.com/cas/520-33-2_894795.html) isomer | Dihydroflavonoid |
| 58 | 9.52 | C_27_H_30_O_15_ | 595.1660 | -0.50 | 449.1113[M+H-Rha]^+^,  287.0553[M+H-Rha-Glc]^+^ | [Kaempferol-3-O-rutinoside](https://www.chemsrc.com/cas/17297-56-2_89274.html) isomer | Flavonol |
| 59 | 9.57 | C_27_H_32_O_14_ | 581.1860 | -1.72 | 273.0760[M+H-Rha-Glc]^+^ | Narirutin [1, 4] | Dihydroflavonoid |
| 60 | 9.57 | C_21_H_22_O_10_ | 435.1285 | -1.38 | 273.0760[M+H-Glc]^+^ | Naringenin-7-O-glucoside isomer | Dihydroflavonoid |
| 61 | 9.57 | C_15_H_12_O_5_ | 273.0757 | -2.20 | 179.0350[M+H-C_6_H_6_O]^+^,  153.0183[M+H-C_8_H_8_O]^+^,  147.0441[M+H-C_6_H_6_O_3_]^+^,  119.0491[M+H-C_7_H_6_O_4_]^+^ | Naringenin isomer | Dihydroflavonoid |
| 62 | 9.59 | C_27_H_30_O_14_ | 579.1709 | -0.86 | 433.1121[M+H-Rha]^+^,  271.0602[M+H-Rha-Glc]^+^ | [Rhoifolin](https://www.chemsrc.com/cas/17306-46-6_141870.html) isomer | Flavone |
| 63 | 9.70 | C_19_H_18_O_8_ | 375.1083 | 0.80 | 360.0849[M+H-CH_3_]^+^,  345.0610[M+H-2CH_3_]^+^,  327.0666[M+H-2CH_3_-H_2_O]^+^,  302.0416[M+H-3CH_3_-CO]^+^ | Quercetagetin-3,7,3′,4′-tetramethyl ether isomer | Flavonol |
| 64 | 9.71 | C_16_H_12_O_6_ | 301.0708 | -1.33 | 286.0470[M+H-CH_3_]^+^,  258.0528[M+H-CH_3_-CO]^+^ | Tectorigenin isomer | Flavone |
| 65 | 9.87 | C_16_H_12_O_6_ | 301.0706 | -1.99 | 286.0475[M+H-CH_3_]^+^,  258.0521[M+H-CH_3_-CO]^+^ | Tectorigenin isomer | Flavone |
| 66 | 9.89 | C_27_H_30_O_14_ | 579.1709 | -0.86 | 433.1120[M+H-Rha]^+^,  271.0604[M+H-Rha-Glc]^+^ | [Rhoifolin](https://www.chemsrc.com/cas/17306-46-6_141870.html) *^a^* [8] | Flavone |
| 67 | 9.92 | C_27_H_32_O_14_ | 581.1860 | -1.72 | 273.0759[M+H-Rha-Glc]^+^ | [Naringin](https://www.chemsrc.com/cas/10236-47-2_402113.html) *^a^* [1] | Dihydroflavonoid |
| 68 | 9.92 | C_15_H_12_O_5_ | 273.0758 | -1.83 | 179.0343[M+H-C_6_H_6_O]^+^,  153.0182[M+H-C_8_H_8_O]^+^,  147.0441[M+H-C_6_H_6_O_3_]^+^,  119.0490[M+H-C_7_H_6_O_4_]^+^ | Naringenin *^a^* [4] | Dihydroflavonoid |
| 69 | 9.93 | C_28_H_34_O_15_ | 611.1970 | -0.98 | 303.0861[M+H-Rha-Glc]^+^ | [Hesperidin](https://www.chemsrc.com/cas/520-26-3_402114.html) isomer/[Neohesperidin](https://www.chemsrc.com/cas/13241-33-3_402164.html) isomer | Dihydroflavonoid |
| 70 | 9.93 | C_16_H_14_O_6_ | 303.0863 | -1.98 | 285.0766[M+H-H_2_O]^+^,  177.0549[M+H-C_6_H_6_O_3_]^+^,  153.0183[M+H-C_9_H_10_O_2_]^+^,  149.0594[M+H-C_9_H_6_O_4_]^+^ | [Hesperetin](https://www.chemsrc.com/cas/520-33-2_894795.html) isomer | Dihydroflavonoid |
| 71 | 9.99 | C_21_H_22_O_10_ | 435.1285 | -1.38 | 273.0761[M+H-Glc]^+^ | Naringenin-7-O-glucoside *^a^* [9] | Dihydroflavonoid |
| 72 | 10.04 | C_28_H_34_O_15_ | 611.1972 | -0.65 | 303.0866[M+H-Rha-Glc]^+^ | [Hesperidin](https://www.chemsrc.com/cas/520-26-3_402114.html) isomer/[Neohesperidin](https://www.chemsrc.com/cas/13241-33-3_402164.html) isomer | Dihydroflavonoid |
| 73 | 10.07 | C_16_H_14_O_6_ | 303.0863 | -1.98 | 285.0765[M+H-H_2_O]^+^,  177.0545[M+H-C_6_H_6_O_3_]^+^,  153.0182[M+H-C_9_H_10_O_2_]^+^,  149.0593[M+H-C_9_H_6_O_4_]^+^ | [Hesperetin](https://www.chemsrc.com/cas/520-33-2_894795.html) isomer | Dihydroflavonoid |
| 74 | 10.08 | C_27_H_32_O_15_ | 597.1818 | -0.17 | 289.0715[M+H-Rha-Glc]^+^ | [Eriocitrin](https://www.chemsrc.com/cas/13463-28-0_122092.html) isomer | Dihydroflavonoid |
| 75 | 10.08 | C_21_H_22_O_10_ | 435.1287 | -0.92 | 273.0744[M+H-Glc]^+^ | Naringenin-7-O-glucoside isomer | Dihydroflavonoid |
| 76 | 10.16 | C_15_H_12_O_5_ | 273.0758 | -1.83 | 179.0338[M+H-C_6_H_6_O]^+^,  153.0182[M+H-C_8_H_8_O]^+^,  147.0441[M+H-C_6_H_6_O_3_]^+^,  119.0493[M+H-C_7_H_6_O_4_]^+^ | Naringenin isomer | Dihydroflavonoid |
| 77 | 10.17 | C_21_H_22_O_10_ | 435.1287 | -0.92 | 273.0768[M+H-Glc]^+^ | Naringenin-7-O-glucoside isomer | Dihydroflavonoid |
| 78 | 10.31 | C_28_H_34_O_15_ | 611.1970 | -0.98 | 303.0863[M+H-Rha-Glc]^+^ | [Hesperidin](https://www.chemsrc.com/cas/520-26-3_402114.html) *^a^* [1] | Dihydroflavonoid |
| 79 | 10.31 | C_16_H_14_O_6_ | 303.0863 | -1.98 | 285.0754[M+H-H_2_O]^+^,  177.0546[M+H-C_6_H_6_O_3_]^+^,  153.0182[M+H-C_9_H_10_O_2_]^+^,  149.0597[M+H-C_9_H_6_O_4_]^+^ | [Hesperetin](https://www.chemsrc.com/cas/520-33-2_894795.html) isomer | Dihydroflavonoid |
| 80 | 10.35 | C_16_H_12_O_6_ | 301.0708 | -1.33 | 286.0482[M+H-CH_3_]^+^,  258.0528[M+H-CH_3_-CO]^+^ | Tectorigenin isomer | Flavone |
| 81 | 10.36 | C_19_H_18_O_8_ | 375.1072 | -2.13 | 360.0858[M+H-CH_3_]^+^,  345.0618[M+H-2CH_3_]^+^ | Quercetagetin-3,7,3′,4′-tetramethyl ether isomer | Flavonol |
| 82 | 10.72 | C_28_H_34_O_15_ | 611.1972 | -0.65 | 303.0860[M+H-Rha-Glc]^+^ | [Neohesperidin](https://www.chemsrc.com/cas/13241-33-3_402164.html) *^a^* [1] | Dihydroflavonoid |
| 83 | 10.72 | C_16_H_14_O_6_ | 303.0863 | -1.98 | 285.0768[M+H-H_2_O]^+^,  177.0547[M+H-C_6_H_6_O_3_]^+^,  153.0182[M+H-C_9_H_10_O_2_]^+^,  149.0595[M+H-C_9_H_6_O_4_]^+^ | [Hesperetin](https://www.chemsrc.com/cas/520-33-2_894795.html) isomer | Dihydroflavonoid |
| 84 | 10.81 | C_27_H_32_O_14_ | 581.1864 | -1.03 | 273.0757[M+H-Rha-Glc]^+^ | [Naringin](https://www.chemsrc.com/cas/10236-47-2_402113.html) isomer | Dihydroflavonoid |
| 85 | 10.95 | C_16_H_14_O_6_ | 303.0863 | -1.98 | 285.0769[M+H-H_2_O]^+^,  177.0546[M+H-C_6_H_6_O_3_]^+^,  153.0182[M+H-C_9_H_10_O_2_]^+^,  149.0597[M+H-C_9_H_6_O_4_]^+^ | [Hesperetin](https://www.chemsrc.com/cas/520-33-2_894795.html) isomer | Dihydroflavonoid |
| 86 | 10.96 | C_28_H_34_O_15_ | 611.1970 | -0.98 | 303.0863[M+H-Rha-Glc]^+^ | [Hesperidin](https://www.chemsrc.com/cas/520-26-3_402114.html) isomer/[Neohesperidin](https://www.chemsrc.com/cas/13241-33-3_402164.html) isomer | Dihydroflavonoid |
| 87 | 11.17 | C_16_H_14_O_6_ | 303.0863 | -1.98 | 285.0745[M+H-H_2_O]^+^,  177.0548[M+H-C_6_H_6_O_3_]^+^,  153.0181[M+H-C_9_H_10_O_2_]^+^,  149.0597[M+H-C_9_H_6_O_4_]^+^ | [Hesperetin](https://www.chemsrc.com/cas/520-33-2_894795.html) isomer | Dihydroflavonoid |
| 88 | 11.43 | C_19_H_18_O_8_ | 375.1071 | -2.40 | 360.0852[M+H-CH_3_]^+^,  345.0635[M+H-2CH_3_]^+^,  330.0374[M+H-3CH_3_]^+^,  327.0513[M+H-2CH_3_-H_2_O]^+^,  317.0679[M+H-2CH_3_-CO]^+^,  302.0415[M+H-3CH_3_-CO]^+^,  299.0550[M+H-2CH_3_-CO-H_2_O]^+^,  271.0608[M+H-2CH_3_-CO-2H_2_O]^+^ | Quercetagetin-3,7,3′,4′-tetramethyl ether isomer | Flavonol |
| 89 | 11.68 | C_28_H_34_O_15_ | 611.1972 | -0.65 | 303.0862[M+H-Rha-Glc]^+^ | [Hesperidin](https://www.chemsrc.com/cas/520-26-3_402114.html) isomer/[Neohesperidin](https://www.chemsrc.com/cas/13241-33-3_402164.html) isomer | Dihydroflavonoid |
| 90 | 12.01 | C_27_H_30_O_14_ | 579.1709 | -0.86 | 271.0588[M+H-Rha-Glc]^+^ | [Rhoifolin](https://www.chemsrc.com/cas/17306-46-6_141870.html) isomer | Flavone |
| 91 | 12.56 | C_20_H_20_O_9_ | 405.1180 | -1.48 | 390.0949[M+H-CH_3_]^+^,  375.0712[M+H-2CH_3_]^+^,  372.0837[M+H-CH_3_-H_2_O]^+^,  357.0609[M+H-2CH_3_-H_2_O]^+^,  347.0763[M+H-2CH_3_-CO]^+^,  332.0530[M+H-3CH_3_-CO]^+^,  329.0659[M+H-2CH_3_-H_2_O-CO]^+^,  317.0283[M+H-4CH_3_-CO]^+^,  304.0592[M+H-3CH_3_-2CO]^+^,  301.0703[M+H-2CH_3_-H_2_O-2CO]^+^,  289.0359[M+H-4CH_3_-2CO]^+^ | 5,4´-Dihydroxy-3,6,7,8,3´-pentamethoxyflavone isomer | Flavonol |
| 92 | 12.69 | C_20_H_20_O_9_ | 405.1180 | -1.48 | 390.0944[M+H-CH_3_]^+^,  375.0710[M+H-2CH_3_]^+^,  372.0840[M+H-CH_3_-H_2_O]^+^,  357.0608[M+H-2CH_3_-H_2_O]^+^,  347.0761[M+H-2CH_3_-CO]^+^,  332.0526[M+H-3CH_3_-CO]^-^,  329.0654[M+H-2CH_3_-H_2_O-CO]^+^,  317.0299[M+H-4CH_3_-CO]^+^,  304.0570[M+H-3CH_3_-2CO]^+^,  301.0710[M+H-2CH_3_-H_2_O-2CO]^+^,  289.0346[M+H-4CH_3_-2CO]^+^ | 5,4´-Dihydroxy-3,6,7,8,3´-pentamethoxyflavone isomer | Flavonol |
| 93 | 12.88 | C_19_H_18_O_8_ | 375.1074 | -1.60 | 360.0858[M+H-CH_3_]^+^,  345.0615[M+H-2CH_3_]^+^,  330.0339[M+H-3CH_3_]^+^,  327.0516[M+H-2CH_3_-H_2_O]^+^,  317.0676[M+H-2CH_3_-CO]^+^,  302.0415[M+H-3CH_3_-CO]^+^,  299.0539[M+H-2CH_3_-CO-H_2_O]^+^ | Quercetagetin-3,7,3′,4′-tetramethyl ether isomer | Flavonol |
| 94 | 13.38 | C_20_H_20_O_9_ | 405.1182 | -0.99 | 390.0943[M+H-CH_3_]^+^,  375.0709[M+H-2CH_3_]^+^,  372.0858[M+H-CH_3_-H_2_O]^+^,  357.0610[M+H-2CH_3_-H_2_O]^+^,  347.0762[M+H-2CH_3_-CO]^+^,  332.0497[M+H-3CH_3_-CO]^+^,  329.0664[M+H-2CH_3_-H_2_O-CO]^+^,  317.0267[M+H-4CH_3_-CO]^-^ | 5,4´-Dihydroxy-3,6,7,8,3´-pentamethoxyflavone isomer | Flavonol |
| 95 | 13.39 | C_19_H_18_O_8_ | 375.1072 | -2.13 | 360.0845[M+H-CH_3_]^+^,  345.0613[M+H-2CH_3_]^+^,  330.0367[M+H-3CH_3_]^+^,  327.0510[M+H-2CH_3_-H_2_O]^+^ | Quercetagetin-3,7,3′,4′-tetramethyl ether isomer | Flavonol |
| 96 | 13.75 | C_20_H_20_O_9_ | 405.1180 | -1.48 | 390.0944[M+H-CH_3_]^+^,  375.0714[M+H-2CH_3_]^+^,  372.0845[M+H-CH_3_-H_2_O]^+^,  357.0595[M+H-2CH_3_-H_2_O]^+^,  347.0761[M+H-2CH_3_-CO]^+^,  332.0528[M+H-3CH_3_-CO]^+^,  329.0664[M+H-2CH_3_-H_2_O-CO]^+^,  317.0278[M+H-4CH_3_-CO]^+^,  304.0557[M+H-3CH_3_-2CO]^+^,  301.0724[M+H-2CH_3_-H_2_O-2CO]^+^,  289.0343[M+H-4CH_3_-2CO]^-^ | 5,4´-Dihydroxy-3,6,7,8,3´-pentamethoxyflavone isomer | Flavonol |
| 97 | 14.06 | C_20_H_20_O_9_ | 405.1181 | -1.23 | 390.0943[M+H-CH_3_]^-^,  375.0710[M+H-2CH_3_]^+^,  372.0846[M+H-CH_3_-H_2_O]^+^,  357.0600[M+H-2CH_3_-H_2_O]^+^,  347.0760[M+H-2CH_3_-CO]^+^,  332.0528[M+H-3CH_3_-CO]^+^,  329.0653[M+H-2CH_3_-H_2_O-CO]^+^,  317.0289[M+H-4CH_3_-CO]^+^,  304.0575[M+H-3CH_3_-2CO]^+^,  301.0709[M+H-2CH_3_-H_2_O-2CO]^+^,  289.0327[M+H-4CH_3_-2CO]^+^ | 5,4´-Dihydroxy-3,6,7,8,3´-pentamethoxyflavone isomer | Flavonol |
| 98 | 14.13 | C_19_H_18_O_7_ | 359.1125 | -1.67 | 344.0876[M+H-CH_3_]^+^,  329.0656[M+H-2CH_3_]^+^ | 5-Hydroxy-3,7,3',4'-tetramethoxyflavone isomer | Flavonol |
| 99 | 14.14 | C_21_H_22_O_9_ | 419.1333 | -2.15 | 404.1100[M+H-CH_3_]^+^,  389.0865[M+H-2CH_3_]^+^,  386.1001[M+H-CH_3_-H_2_O]^+^,  371.0760[M+H-2CH_3_-H_2_O]^+^,  361.0919[M+H-2CH_3_-CO]^+^,  346.0681[M+H-3CH_3_-CO]^+^,  328.0575[M+H-3CH_3_-CO-H_2_O]^+^,  313.0349[M+H-4CH_3_-CO-H_2_O]^+^ | 8-Hydroxy-3,5,6,7,3′,4′-hexamethoxyflavone isomer | Flavonol |
| 100 | 14.28 | C_19_H_18_O_8_ | 375.1075 | -2.13 | 360.0854[M+H-CH_3_]^+^,  345.0599[M+H-2CH_3_]^+^ | Quercetagetin-3,7,3′,4′-tetramethyl ether isomer | Flavonol |
| 101 | 14.45 | C_20_H_20_O_9_ | 405.1180 | -1.48 | 390.0941[M+H-CH_3_]^+^,  375.0709[M+H-2CH_3_]^+^,  372.0847[M+H-CH_3_-H_2_O]^+^,  357.0604[M+H-2CH_3_-H_2_O]^+^,  347.0761[M+H-2CH_3_-CO]^+^,  332.0526[M+H-3CH_3_-CO]^+^,  329.0653[M+H-2CH_3_-H_2_O-CO]^+^,  317.0292[M+H-4CH_3_-CO]^+^,  304.0566[M+H-3CH_3_-2CO]^+^,  289.0344[M+H-4CH_3_-2CO]^+^ | 5,4´-Dihydroxy-3,6,7,8,3´-pentamethoxyflavone isomer | Flavonol |
| 102 | 14.52 | C_19_H_18_O_7_ | 359.1126 | -1.39 | 344.0880[M+H-CH_3_]^+^,  329.0654[M+H-2CH_3_]^+^,  326.0790[M+H-CH_3_-H_2_O]^+^,  298.0833[M+H-CH_3_-H_2_O-CO]^+^,  283.0602[M+H-2CH_3_-H_2_O-CO]^+^ | 5-Hydroxy-3,7,3',4'-tetramethoxyflavone isomer | Flavonol |
| 103 | 14.97 | C_19_H_18_O_7_ | 359.1126 | -1.39 | 344.0880[M+H-CH_3_]^+^,  329.0657[M+H-2CH_3_]^+^,  326.0779[M+H-CH_3_-H_2_O]^+^,  298.0829[M+H-CH_3_-H_2_O-CO]^+^,  283.0589[M+H-2CH_3_-H_2_O-CO]^+^ | 5-Hydroxy-3,7,3',4'-tetramethoxyflavone isomer | Flavonol |
| 104 | 15.23 | C_19_H_18_O_7_ | 359.1125 | -1.67 | 344.0856[M+H-CH_3_]^+^,  329.0627[M+H-2CH_3_]^+^,  326.0783[M+H-CH_3_-H_2_O]^+^,  298.0807[M+H-CH_3_-H_2_O-CO]^+^ | 5-Hydroxy-3,7,3',4'-tetramethoxyflavone isomer | Flavonol |
| 105 | 15.29 | C_16_H_12_O_6_ | 301.0706 | -1.99 | 286.0471[M+H-CH_3_]^+^,  258.0525[M+H-CH_3_-CO]^+^ | Tectorigenin isomer | Flavone |
| 106 | 15.34 | C_18_H_16_O_7_ | 345.0969 | -1.45 | 330.0727[M+H-CH_3_]^+^,  315.0493[M+H-2CH_3_]^+^,  287.0559[M+H-2CH_3_-CO]^+^ | Dihydroxy-trimethoxyflavone isomer | Flavonol |
| 107 | 15.43 | C_19_H_18_O_8_ | 375.1074 | -1.60 | 360.0837[M+H-CH_3_]^+^,  345.0590[M+H-2CH_3_]^+^ | Quercetagetin-3,7,3′,4′-tetramethyl ether isomer | Flavonol |
| 108 | 15.51 | C_16_H_14_O_6_ | 303.0863 | -1.98 | 285.0757[M+H-H_2_O]^+^,  177.0547[M+H-C_6_H_6_O_3_]^+^,  153.0182[M+H-C_9_H_10_O_2_]^+^,  149.0595[M+H-C_9_H_6_O_4_]^+^ | [Hesperetin](https://www.chemsrc.com/cas/520-33-2_894795.html) *^a^* [4] | Dihydroflavonoid |
| 109 | 15.54 | C_20_H_20_O_9_ | 405.1180 | -1.48 | 390.0929[M+H-CH_3_]^+^,  375.0694[M+H-2CH_3_]^+^,  372.0829[M+H-CH_3_-H_2_O]^+^,  357.0592[M+H-2CH_3_-H_2_O]^+^,  347.0748[M+H-2CH_3_-CO]^+^,  332.0511[M+H-3CH_3_-CO]^+^,  329.0649[M+H-2CH_3_-H_2_O-CO]^+^,  317.0280[M+H-4CH_3_-CO]^+^,  289.0344[M+H-4CH_3_-2CO]^+^ | 5,4´-Dihydroxy-3,6,7,8,3´-pentamethoxyflavone isomer | Flavonol |
| 110 | 15.79 | C_21_H_22_O_9_ | 419.1332 | -2.39 | 404.1103[M+H-CH_3_]^+^,  389.0865[M+H-2CH_3_]^+^,  386.1002[M+H-CH_3_-H_2_O]^+^,  371.0765[M+H-2CH_3_-H_2_O]^+^,  361.0916[M+H-2CH_3_-CO]^+^,  346.0683[M+H-3CH_3_-CO]^+^,  328.0576[M+H-3CH_3_-CO-H_2_O]^+^,  313.0348[M+H-4CH_3_-CO-H_2_O]^+^ | 8-Hydroxy-3,5,6,7,3′,4′-hexamethoxyflavone isomer | Flavonol |
| 111 | 15.80 | C_19_H_18_O_7_ | 359.1126 | -1.39 | 344.0882[M+H-CH_3_]^+^,  329.0663[M+H-2CH_3_]^+^,  298.0844[M+H-CH_3_-H_2_O-CO]^+^ | 5-Hydroxy-3,7,3',4'-tetramethoxyflavone isomer | Flavonol |
| 112 | 15.97 | C_20_H_20_O_8_ | 389.1231 | -1.28 | 374.0985[M+H-CH_3_]^+^,  359.0764[M+H-2CH_3_]^+^,  356.0888[M+H-CH_3_-H_2_O]^+^,  341.0657[M+H-2CH_3_-H_2_O]^+^,  331.0811[M+H-2CH_3_-CO]^+^,  328.0950[M+H-CH_3_-H_2_O-CO]^+^,  313.0711[M+H-2CH_3_-H_2_O-CO]^+^ | [5-Demethylnobiletin](https://www.chemsrc.com/cas/2174-59-6_964689.html) isomer | Flavone |
| 113 | 15.99 | C_19_H_18_O_7_ | 359.1125 | -1.67 | 344.0886[M+H-CH_3_]^+^,  329.0657[M+H-2CH_3_]^+^,  326.0788[M+H-CH_3_-H_2_O]^+^,  298.0834[M+H-CH_3_-H_2_O-CO]^+^,  283.0602[M+H-2CH_3_-H_2_O-CO]^+^ | 5-Hydroxy-3,7,3',4'-tetramethoxyflavone isomer | Flavonol |
| 114 | 16.14 | C_20_H_20_O_9_ | 405.1183 | -0.74 | 390.0933[M+H-CH_3_]^+^,  375.0714[M+H-2CH_3_]^+^,  372.0839[M+H-CH_3_-H_2_O]^+^,  357.0607[M+H-2CH_3_-H_2_O]^+^,  347.0763[M+H-2CH_3_-CO]^+^,  332.0533[M+H-3CH_3_-CO]^+^,  329.0663[M+H-2CH_3_-H_2_O-CO]^+^,  317.0288[M+H-4CH_3_-CO]^+^,  301.0709[M+H-2CH_3_-H_2_O-2CO]^+^ | 5,4´-Dihydroxy-3,6,7,8,3´-pentamethoxyflavone isomer | Flavonol |
| 115 | 16.17 | [C](https://pubchem.ncbi.nlm.nih.gov/#query=C20H22O7)_20_H_22_O_7_ | 375.1440 | -1.07 | 211.0600[M+H-C_10_H_12_O_2_]^+^,  196.0365[M+H-C_10_H_12_O_2_-CH_3_]^+^,  191.0698[M+H-C_9_H_12_O_4_]^+^,  168.0417[M+H-C_10_H_12_O_2_-CH_3_-CO]^+^,  150.0311[M+H-C_10_H_12_O_2_-CH_3_-CO-H_2_O]^+^ | 2´-Hydroxy-3,4,4´,5´,6´-pentamethoxychalcone isomer | Chalcone |
| 116 | 16.23 | C_16_H_12_O_6_ | 301.0704 | -2.66 | 286.0483[M+H-CH_3_]^+^ | Tectorigenin isomer | Flavone |
| 117 | 16.41 | C_20_H_20_O_9_ | 405.1180 | -1.48 | 390.0950[M+H-CH_3_]^+^,  375.0712[M+H-2CH_3_]^+^,  372.0839[M+H-CH_3_-H_2_O]^+^,  357.0603[M+H-2CH_3_-H_2_O]^+^,  347.0761[M+H-2CH_3_-CO]^+^,  332.0529[M+H-3CH_3_-CO]^+^,  329.0645[M+H-2CH_3_-H_2_O-CO]^+^,  317.0286[M+H-4CH_3_-CO]^+^,  289.0347[M+H-4CH_3_-2CO]^+^ | 5,4´-Dihydroxy-3,6,7,8,3´-pentamethoxyflavone isomer | Flavonol |
| 118 | 16.73 | C_19_H_18_O_8_ | 375.1075 | -2.13 | 360.0852[M+H-CH_3_]^+^ | Quercetagetin-3,7,3′,4′-tetramethyl ether isomer | Flavonol |
| 119 | 16.77 | C_17_H_14_O_7_ | 331.0813 | -1.51 | 316.0580[M+H-CH_3_]^+^,  301.0350[M+H-2CH_3_]^+^,  273.0425[M+H-2CH_3_-CO]^+^,  253.3105[M+H-2CH_3_-CO-H_2_O]^+^,  245.0452[M+H-2CH_3_-2CO]^+^ | [Tricin](https://www.chemsrc.com/cas/520-32-1_183504.html) [10] | Flavone |
| 120 | 16.78 | C_20_H_20_O_8_ | 389.1230 | -1.54 | 374.0995[M+H-CH_3_]^+^,  359.0762[M+H-2CH_3_]^+^,  356.0806[M+H-CH_3_-H_2_O]^+^,  341.0650[M+H-2CH_3_-H_2_O]^+^,  331.0808[M+H-2CH_3_-CO]^+^,  313.0705[M+H-2CH_3_-H_2_O-CO]^+^ | [5-Demethylnobiletin](https://www.chemsrc.com/cas/2174-59-6_964689.html) isomer | Flavone |
| 121 | 16.82 | C_20_H_22_O_8_ | 391.1403 | 2.56 | 376.1060[M+H-CH_3_]^+^,  361.0820[M+H-2CH_3_]^+^ | 5-Hydroxy-6,7,8,3´,4´-pentamethoxyflavanone isomer | Dihydroflavonoid |
| 122 | 17.04 | C_20_H_20_O_7_ | 373.1279 | -2.14 | 358.1051[M+H-CH_3_]^+^,  343.0811[M+H-2CH_3_]^+^,  315.0866[M+H-2CH_3_-CO]^+^ | [Isosinensetin](https://www.chemsrc.com/cas/17290-70-9_964698.html) *^a^* [1, 4] | Flavone |
| 123 | 17.05 | C_19_H_18_O_7_ | 359.1126 | -1.39 | 344.0891[M+H-CH_3_]^+^,  329.0665[M+H-2CH_3_]^+^,  283.0608[M+H-2CH_3_-H_2_O-CO]^+^ | 5-Hydroxy-3,7,3',4'-tetramethoxyflavone isomer | Flavonol |
| 124 | 17.32 | C_20_H_22_O_8_ | 391.1387 | -1.53 | 376.1013[M+H-CH_3_]^+^,  361.0827[M+H-2CH_3_]^+^,  241.0703[M+H-5OCH_2_]^+^ | 5-Hydroxy-6,7,8,3´,4´-pentamethoxyflavanone isomer | Dihydroflavonoid |
| 125 | 17.58 | C_20_H_20_O_8_ | 389.1230 | -1.54 | 374.0994[M+H-CH_3_]^+^,  359.0762[M+H-2CH_3_]^+^,  341.0657[M+H-2CH_3_-H_2_O]^+^,  331.0768[M+H-2CH_3_-CO]^+^,  328.0939[M+H-CH_3_-H_2_O-CO]^+^,  313.0703[M+H-2CH_3_-H_2_O-CO]^+^ | [5-Demethylnobiletin](https://www.chemsrc.com/cas/2174-59-6_964689.html) isomer | Flavone |
| 126 | 17.77 | C_20_H_22_O_8_ | 391.1387 | -1.53 | 376.1175[M+H-CH_3_]^+^,  361.0817[M+H-2CH_3_]^+^,  241.0706[M+H-5OCH_2_]^+^ | 5-Hydroxy-6,7,8,3´,4´-pentamethoxyflavanone [4] | Dihydroflavonoid |
| 127 | 17.77 | C_21_H_22_O_9_ | 419.1333 | -2.15 | 404.1104[M+H-CH_3_]^+^,  389.0867[M+H-2CH_3_]^+^,  386.0997[M+H-CH_3_-H_2_O]^+^,  371.0760[M+H-2CH_3_-H_2_O]^+^,  361.0916[M+H-2CH_3_-CO]^+^,  346.0679[M+H-3CH_3_-CO]^+^,  328.0573[M+H-3CH_3_-CO-H_2_O]^+^,  313.0349[M+H-4CH_3_-CO-H_2_O]^+^ | 8-Hydroxy-3,5,6,7,3′,4′-hexamethoxyflavone isomer | Flavonol |
| 128 | 18.13 | C_19_H_18_O_6_ | 343.1179 | -0.87 | 328.0934[M+H-CH_3_]^+^,  313.0703[M+H-2CH_3_]^+^,  299.0912[M+H-CH_3_-CHO]^+^,  285.0750[M+H-2CH_3_-CO]^+^,  257.0790[M+H-2CH_3_-2CO]^+^ | 4´,5,6,7-Tetramethoxyflavone isomer | Flavonol |
| 129 | 18.18 | C_21_H_22_O_8_ | 403.1384 | -2.23 | 388.1146[M+H-CH_3_]^+^,  373.0919[M+H-2CH_3_]^+^,  355.0811[M+H-2CH_3_-H_2_O]^+^,  327.0870[M+H-2CH_3_-H_2_O-CO]^+^ | [Nobiletin](https://www.chemsrc.com/cas/478-01-3_122104.html) isomer | Flavone |
| 130 | 18.33 | C_20_H_20_O_9_ | 405.1184 | -0.49 | 390.0931[M+H-CH_3_]^+^,  375.0683[M+H-2CH_3_]^+^,  372.0855[M+H-CH_3_-H_2_O]^+^,  357.0584[M+H-2CH_3_-H_2_O]^+^,  347.0756[M+H-2CH_3_-CO]^+^,  332.0517M+H-3CH_3_-CO]^+^,  329.0661[M+H-2CH_3_-H_2_O-CO]^+^,  317.0284[M+H-4CH_3_-CO]^+^ | 5,4´-Dihydroxy-3,6,7,8,3´-pentamethoxyflavone isomer | Flavonol |
| 131 | 18.44 | C_18_H_16_O_7_ | 345.0970 | -1.16 | 330.0751[M+H-CH_3_]^+^ | Dihydroxy-trimethoxyflavone isomer | Flavonol |
| 132 | 18.57 | C_21_H_22_O_9_ | 419.1332 | -2.39 | 404.1104[M+H-CH_3_]^+^,  389.0868[M+H-2CH_3_]^+^,  386.0995[M+H-CH_3_-H_2_O]^+^,  371.0767[M+H-2CH_3_-H_2_O]^+^,  361.0930[M+H-2CH_3_-CO]^+^,  346.0685[M+H-3CH_3_-CO]^+^,  328.0560[M+H-3CH_3_-CO-H_2_O]^+^,  313.0349[M+H-4CH_3_-CO-H_2_O]^+^ | 8-Hydroxy-3,5,6,7,3′,4′-hexamethoxyflavone isomer | Flavonol |
| 133 | 18.68 | C_20_H_20_O_7_ | 373.1279 | -2.14 | 358.0966[M+H-CH_3_]^+^,  343.0811[M+H-2CH_3_]^+^,  315.0862[M+H-2CH_3_-CO]^+^ | [Sinensetin](https://www.chemsrc.com/cas/2306-27-6_141840.html) *^a^* [4] | Flavone |
| 134 | 18.73 | C_19_H_20_O_6_ | 345.1333 | -1.45 | 211.0600[M+H-C_9_H_10_O]^+^,  196.0364[M+H-C_9_H_10_O-CH_3_]^+^,  168.0421[M+H-C_9_H_10_O-CH_3_-CO]^+^,  150.0311[M+H-C_9_H_10_O-CH_3_-CO-H_2_O]^+^ | [5,6,7,4'-Tetramethoxyflavanone](https://www.chemsrc.com/cas/72943-90-9_1680019.html) isomer | Dihydroflavonoid |
| 135 | 18.98 | C_20_H_20_O_8_ | 389.1228 | -2.06 | 374.0989[M+H-CH_3_]^+^,  359.0763[M+H-2CH_3_]^+^,  331.0815[M+H-2CH_3_-CO]^+^ | [5-Demethylnobiletin](https://www.chemsrc.com/cas/2174-59-6_964689.html) isomer | Flavone |
| 136 | 19.02 | C_19_H_18_O_7_ | 359.1125 | -1.67 | 344.0899[M+H-CH_3_]^+^,  329.0654[M+H-2CH_3_]^+^,  326.0791[M+H-CH_3_-H_2_O]^+^,  283.0607[M+H-2CH_3_-H_2_O-CO]^+^ | 5-Hydroxy-3,7,3',4'-tetramethoxyflavone isomer | Flavonol |
| 137 | 19.03 | C_18_H_16_O_7_ | 345.0969 | -1.45 | 330.0726[M+H-CH_3_]^+^,  315.0493[M+H-2CH_3_]^+^ | Dihydroxy-trimethoxyflavone isomer | Flavonol |
| 138 | 19.11 | C_19_H_18_O_6_ | 343.1178 | -1.17 | 328.0945[M+H-CH_3_]^+^,  313.0705[M+H-2CH_3_]^+^,  299.0891[M+H-CH_3_-CHO]^+^,  285.0756[M+H-2CH_3_-CO]^+^,  257.0820[M+H-2CH_3_-2CO]^+^ | 4´,5,6,7-Tetramethoxyflavone isomer | Flavonol |
| 139 | 19.24 | C_20_H_20_O_9_ | 405.1180 | -1.48 | 390.0945[M+H-CH_3_]^+^,  375.0709[M+H-2CH_3_]^+^,  372.0839[M+H-CH_3_-H_2_O]^+^,  357.0593[M+H-2CH_3_-H_2_O]^+^,  347.0760[M+H-2CH_3_-CO]^+^,  332.0532[M+H-3CH_3_-CO]^+^,  329.0655[M+H-2CH_3_-H_2_O-CO]^+^,  317.0320[M+H-4CH_3_-CO]^+^,  289.0342[M+H-4CH_3_-2CO]^+^ | 5,4´-Dihydroxy-3,6,7,8,3´-pentamethoxyflavone isomer | Flavonol |
| 140 | 19.26 | C_20_H_20_O_8_ | 389.1231 | -1.28 | 374.0992[M+H-CH_3_]^+^,  359.0764[M+H-2CH_3_]^+^,  331.0799[M+H-2CH_3_-CO]^+^,  328.0920[M+H-CH_3_-H_2_O-CO]^+^,  313.0706[M+H-2CH_3_-H_2_O-CO]^+^ | [5-Demethylnobiletin](https://www.chemsrc.com/cas/2174-59-6_964689.html) isomer | Flavone |
| 141 | 19.32 | C_20_H_20_O_7_ | 373.1280 | -1.88 | 358.1041[M+H-CH_3_]^+^,  343.0809[M+H-2CH_3_]^+^,  315.0849[M+H-2CH_3_-CO]^+^ | [Isosinensetin](https://www.chemsrc.com/cas/17290-70-9_964698.html) isomer/[Sinensetin](https://www.chemsrc.com/cas/2306-27-6_141840.html) isomer/[Tangeretin](https://www.chemsrc.com/cas/481-53-8_950947.html) isomer | Flavone |
| 142 | 19.37 | [C](https://pubchem.ncbi.nlm.nih.gov/#query=C20H22O7)_20_H_22_O_7_ | 375.1436 | -2.13 | 211.0601[M+H-C_10_H_12_O_2_]^+^,  196.0366[M+H-C_10_H_12_O_2_-CH_3_]^+^,  191.0695[M+H-C_9_H_12_O_4_]^+^,  168.0413[M+H-C_10_H_12_O_2_-CH_3_-CO]^+^,  150.0311[M+H-C_10_H_12_O_2_-CH_3_-CO-H_2_O]^+^ | 2´-Hydroxy-3,4,4´,5´,6´-pentamethoxychalcone | Chalcone |
| 143 | 19.45 | C_19_H_18_O_8_ | 375.1088 | 2.13 | 360.0825[M+H-CH_3_]^+^,  345.0627[M+H-2CH_3_]^+^,  317.0652[M+H-2CH_3_-CO]^+^ | Quercetagetin-3,7,3′,4′-tetramethyl ether isomer | Flavonol |
| 144 | 19.99 | C_21_H_22_O_8_ | 403.1385 | -1.98 | 388.1153[M+H-CH_3_]^+^,  373.0924[M+H-2CH_3_]^+^,  355.0817[M+H-2CH_3_-H_2_O]^+^,  327.0867[M+H-2CH_3_-H_2_O-CO]^+^ | Quercetagetin-3,5,6,7,3′,4′-hexamethyl ether *^a^* | Flavonol |
| 145 | 20.06 | C_18_H_16_O_7_ | 345.0969 | -1.45 | 330.0732[M+H-CH_3_]^+^,  315.0490[M+H-2CH_3_]^+^,  287.0546[M+H-2CH_3_-CO]^+^,  269.0445[M+H-2CH_3_-CO-H_2_O]^+^ | Dihydroxy-trimethoxyflavone isomer | Flavonol |
| 146 | 20.39 | C_19_H_18_O_8_ | 375.1074 | -1.60 | 360.0830[M+H-CH_3_]^+^,  345.0602[M+H-2CH_3_]^+^,  317.0667[M+H-2CH_3_-CO]^+^,  299.0546[M+H-2CH_3_-CO-H_2_O]^+^ | Quercetagetin-3,7,3′,4′-tetramethyl ether isomer | Flavonol |
| 147 | 20.41 | C_18_H_16_O_7_ | 345.0968 | -1.74 | 330.0723[M+H-CH_3_]^+^,  315.0493[M+H-2CH_3_]^+^,  287.0543[M+H-2CH_3_-CO]^+^,  269.0443[M+H-2CH_3_-CO-H_2_O]^+^ | Dihydroxy-trimethoxyflavone | Flavonol |
| 148 | 20.46 | C_21_H_24_O_8_ | 405.1541 | -1.97 | 241.0709[M+H-C_10_H_12_O_2_]^+^,  226.0475[M+H-C_10_H_12_O_2_-CH_3_]^+^,  211.0239[M+H-C_10_H_12_O_2_-2CH_3_]^+^,  208.0367[M+H-C_10_H_12_O_2_-CH_3_-H_2_O]^+^,  198.0520[M+H-C_10_H_12_O_2_-CH_3_-CO]^+^,191.0704[M+H-C_10_H_14_O_5_]^+^,  183.0290[M+H-C_10_H_12_O_2_-2CH_3_-CO]^+^ | 2´-Hydroxy-3,4,3´,4´,5´,6´-hexamethoxychalcone | Chalcone |
| 149 | 20.54 | C_19_H_18_O_8_ | 375.1075 | -2.13 | 360.0822[M+H-CH_3_]^+^,  345.0604[M+H-2CH_3_]^+^,  317.0648[M+H-2CH_3_-CO]^+^,  299.0558[M+H-2CH_3_-CO-H_2_O]^+^,  271.0614[M+H-2CH_3_-CO-2H_2_O]^+^ | Quercetagetin-3,7,3′,4′-tetramethyl ether isomer | Flavonol |
| 150 | 20.71 | C_20_H_20_O_7_ | 373.1281 | -1.61 | 358.1063[M+H-CH_3_]^+^,  343.0813[M+H-2CH_3_]^+^,  315.0872[M+H-2CH_3_-CO]^+^ | [Isosinensetin](https://www.chemsrc.com/cas/17290-70-9_964698.html) isomer/[Sinensetin](https://www.chemsrc.com/cas/2306-27-6_141840.html) isomer/[Tangeretin](https://www.chemsrc.com/cas/481-53-8_950947.html) isomer | Flavone |
| 151 | 20.72 | C_21_H_22_O_8_ | 403.1383 | -2.48 | 388.1154[M+H-CH_3_]^+^,  373.0917[M+H-2CH_3_]^+^,  355.0814[M+H-2CH_3_-H_2_O]^+^,  327.0867[M+H-2CH_3_-H_2_O-CO]^+^ | [Nobiletin](https://www.chemsrc.com/cas/478-01-3_122104.html) *^a^* [4] | Flavone |
| 152 | 20.81 | C_19_H_18_O_8_ | 375.1079 | -0.27 | 360.0840[M+H-CH_3_]^+^,  345.0585[M+H-2CH_3_]^+^,  327.0498[M+H-2CH_3_-H_2_O]^+^ | Quercetagetin-3,7,3′,4′-tetramethyl ether isomer | Flavonol |
| 153 | 20.92 | C_19_H_18_O_6_ | 343.1179 | -0.87 | 328.0936[M+H-CH_3_]^+^,  313.0705[M+H-2CH_3_]^+^,  299.0910[M+H-CH_3_-CHO]^+^,  285.0754[M+H-2CH_3_-CO]^+^ | 4´,5,6,7-Tetramethoxyflavone *^a^* [1] | Flavonol |
| 154 | 21.04 | C_19_H_18_O_8_ | 375.1075 | -2.13 | 360.0844[M+H-CH_3_]^+^,  345.0608[M+H-2CH_3_]^+^,  330.0372[M+H-3CH_3_]^+^,  327.0503[M+H-2CH_3_-H_2_O]^+^,  317.0658[M+H-2CH_3_-CO]^+^,  302.0431[M+H-3CH_3_-CO]^+^,  299.0546[M+H-2CH_3_-CO-H_2_O]^+^,  271.0620[M+H-2CH_3_-CO-2H_2_O]^+^ | Quercetagetin-3,7,3′,4′-tetramethyl ether | Flavonol |
| 155 | 21.08 | C_20_H_20_O_8_ | 389.1229 | -1.80 | 374.0999[M+H-CH_3_]^+^,  359.0768[M+H-2CH_3_]^+^,  356.0890[M+H-CH_3_-H_2_O]^+^,  341.0661[M+H-2CH_3_-H_2_O]^+^,  331.0815[M+H-2CH_3_-CO]^+^,  328.0940[M+H-CH_3_-H_2_O-CO]^+^,  313.0710[M+H-2CH_3_-H_2_O-CO]^+^ | [5-Demethylnobiletin](https://www.chemsrc.com/cas/2174-59-6_964689.html) isomer | Flavone |
| 156 | 21.54 | C_19_H_18_O_8_ | 375.1075 | -2.13 | 360.0833[M+H-CH_3_]^+^,  345.0602[M+H-2CH_3_]^+^,  330.0360[M+H-3CH_3_]^+^,  327.0498[M+H-2CH_3_-H_2_O]^+^,  317.0649[M+H-2CH_3_-CO]^+^,  302.0423[M+H-3CH_3_-CO]^+^,  299.0546[M+H-2CH_3_-CO-H_2_O]^+^,  271.0604[M+H-2CH_3_-CO-2H_2_O]^+^ | Quercetagetin-3,7,3′,4′-tetramethyl ether isomer | Flavonol |
| 157 | 21.87 | C_19_H_18_O_8_ | 375.1075 | -2.13 | 360.0837[M+H-CH_3_]^+^,  345.0603[M+H-2CH_3_]^+^,  330.0358[M+H-3CH_3_]^+^,  327.0500[M+H-2CH_3_-H_2_O]^+^,  317.0654[M+H-2CH_3_-CO]^+^,  302.0417[M+H-3CH_3_-CO]^+^,  299.0539[M+H-2CH_3_-CO-H_2_O]^+^,  271.0610[M+H-2CH_3_-CO-2H_2_O]^+^ | Quercetagetin-3,7,3′,4′-tetramethyl ether isomer | Flavonol |
| 158 | 22.00 | C_19_H_18_O_6_ | 343.1177 | -1.46 | 328.0941[M+H-CH_3_]^+^,  313.0708[M+H-2CH_3_]^+^,  299.0911[M+H-CH_3_-CHO]^+^,  285.0747[M+H-2CH_3_-CO]^+^ | 4´,5,6,7-Tetramethoxyflavone isomer | Flavonol |
| 159 | 22.01 | C_22_H_24_O_9_ | 433.1489 | -2.31 | 418.1262[M+H-CH_3_]^+^,  403.1025[M+H-2CH_3_]^+^,  400.1153[M+H-CH_3_-H_2_O]^+^,  385.0921[M+H-2CH_3_-H_2_O]^+^ | 3,3′,4′,5,6,7,8-heptamethoxyflavone [4] | Flavonol |
| 160 | 22.11 | C_20_H_20_O_9_ | 405.1181 | -1.23 | 390.0926[M+H-CH_3_]^+^,  375.0701[M+H-2CH_3_]^+^,  372.0855[M+H-CH_3_-H_2_O]^+^,  357.0599[M+H-2CH_3_-H_2_O]^+^,  347.0759[M+H-2CH_3_-CO]^+^,  332.0518[M+H-3CH_3_-CO]^+^,  329.0682[M+H-2CH_3_-H_2_O-CO]^+^ | 5,4´-Dihydroxy-3,6,7,8,3´-pentamethoxyflavone isomer | Flavonol |
| 161 | 22.29 | C_19_H_20_O_6_ | 345.1333 | -1.45 | 303.1200[M+H-C_2_H_2_O]^+^,  211.0600[M+H-C_9_H_10_O]^+^,  196.0366[M+H-C_9_H_10_O-CH_3_]^+^,  168.0417[M+H-C_9_H_10_O-CH_3_-CO]^+^,  150.0311[M+H-C_9_H_10_O-CH_3_-CO-H_2_O]^+^ | [5,6,7,4'-Tetramethoxyflavanone](https://www.chemsrc.com/cas/72943-90-9_1680019.html) | Dihydroflavonoid |
| 162 | 22.32 | C_19_H_18_O_7_ | 359.1125 | -1.67 | 344.0890[M+H-CH_3_]^+^,  326.0786[M+H-CH_3_-H_2_O]^+^,  298.0838[M+H-CH_3_-H_2_O-CO]^+^,  283.0607[M+H-2CH_3_-H_2_O-CO]^+^,  270.0893[M+H-CH_3_-H_2_O-2CO]^+^,  254.0578[M+H-2CH_3_-H_2_O-2CO]^+^,  242.0895[M+H-CH_3_-H_2_O-3CO]^+^ | 5-Hydroxy-3,7,3',4'-tetramethoxyflavone isomer | Flavonol |
| 163 | 22.38 | C_20_H_20_O_9_ | 405.1180 | -1.48 | 390.0942[M+H-CH_3_]^+^,  375.0709[M+H-2CH_3_]^+^,  372.0839[M+H-CH_3_-H_2_O]^+^,  357.0605[M+H-2CH_3_-H_2_O]^+^,  347.0762[M+H-2CH_3_-CO]^+^,  332.0526[M+H-3CH_3_-CO]^+^,  329.0662[M+H-2CH_3_-H_2_O-CO]^+^,  317.0302[M+H-4CH_3_-CO]^+^,  304.0560[M+H-3CH_3_-2CO]^+^,  301.0710[M+H-2CH_3_-H_2_O-2CO]^+^,  289.0355[M+H-4CH_3_-2CO]^+^ | 5,4´-Dihydroxy-3,6,7,8,3´-pentamethoxyflavone isomer | Flavonol |
| 164 | 22.48 | C_20_H_22_O_8_ | 391.1399 | 1.53 | 241.0706[M+H-5OCH_2_]^+^ | 5-Hydroxy-6,7,8,3´,4´-pentamethoxyflavanone isomer | Dihydroflavonoid |
| 165 | 22.58 | C_20_H_20_O_7_ | 373.1281 | -1.61 | 358.1036[M+H-CH_3_]^+^,  343.0813[M+H-2CH_3_]^+^,  315.0860[M+H-2CH_3_-CO]^+^ | [Isosinensetin](https://www.chemsrc.com/cas/17290-70-9_964698.html) isomer/[Sinensetin](https://www.chemsrc.com/cas/2306-27-6_141840.html) isomer/[Tangeretin](https://www.chemsrc.com/cas/481-53-8_950947.html) isomer | Flavone |
| 166 | 22.59 | C_19_H_18_O_8_ | 375.1077 | -0.80 | 360.0856[M+H-CH_3_]^+^,  345.0604[M+H-2CH_3_]^+^,  271.0579[M+H-2CH_3_-CO-2H_2_O]^+^ | Quercetagetin-3,7,3′,4′-tetramethyl ether isomer | Flavonol |
| 167 | 22.65 | C_21_H_22_O_9_ | 419.1333 | -2.15 | 404.1101[M+H-CH_3_]^+^,  389.0866[M+H-2CH_3_]^+^,  386.0996[M+H-CH_3_-H_2_O]^+^,  374.0630[M+H-3CH_3_]^+^,  371.0765[M+H-2CH_3_-H_2_O]^+^,  361.0920[M+H-2CH_3_-CO]^+^,  346.0684[M+H-3CH_3_-CO]^+^,  343.0816[M+H-2CH_3_-CO-H_2_O]^+^,331.0443[M+H-4CH_3_-CO]^+^,  328.0580[M+H-3CH_3_-CO-H_2_O]^+^,  313.0324[M+H-4CH_3_-CO-H_2_O]^+^ | 8-Hydroxy-3,5,6,7,3′,4′-hexamethoxyflavone isomer | Flavonol |
| 168 | 22.82 | C_20_H_20_O_7_ | 373.1278 | -2.41 | 358.1046[M+H-CH_3_]^+^,  343.0811[M+H-2CH_3_]^+^,  315.0861[M+H-2CH_3_-CO]^+^ | [Tangeretin](https://www.chemsrc.com/cas/481-53-8_950947.html) *^a^* [4] | Flavone |
| 169 | 23.15 | C_19_H_18_O_7_ | 359.1125 | -1.67 | 344.0883[M+H-CH_3_]^+^,  326.0788[M+H-CH_3_-H_2_O]^+^,  298.0837[M+H-CH_3_-H_2_O-CO]^+^,  283.0596[M+H-2CH_3_-H_2_O-CO]^+^ | 5-Hydroxy-3,7,3',4'-tetramethoxyflavone isomer | Flavonol |
| 170 | 23.25 | C_19_H_18_O_8_ | 375.1075 | -2.13 | 360.0809[M+H-CH_3_]^+^,  345.0604[M+H-2CH_3_]^+^,  317.0642[M+H-2CH_3_-CO]^+^ | Quercetagetin-3,7,3′,4′-tetramethyl ether isomer | Flavonol |
| 171 | 23.49 | C_20_H_20_O_8_ | 389.1229 | -1.80 | 374.0984[M+H-CH_3_]^+^,  359.0762[M+H-2CH_3_]^+^,  356.0883[M+H-CH_3_-H_2_O]^+^,  341.0650[M+H-2CH_3_-H_2_O]^+^,  331.0811[M+H-2CH_3_-CO]^+^,  328.0919[M+H-CH_3_-H_2_O-CO]^+^,  313.0701[M+H-2CH_3_-H_2_O-CO]^+^ | [5-Demethylnobiletin](https://www.chemsrc.com/cas/2174-59-6_964689.html) isomer | Flavone |
| 172 | 23.65 | C_19_H_18_O_7_ | 359.1125 | -1.67 | 344.0884[M+H-CH_3_]^+^,  326.0789[M+H-CH_3_-H_2_O]^+^,  298.0832[M+H-CH_3_-H_2_O-CO]^+^,  283.0603[M+H-2CH_3_-H_2_O-CO]^+^ | 5-Hydroxy-3,7,3',4'-tetramethoxyflavone isomer | Flavonol |
| 173 | 23.66 | C_20_H_20_O_9_ | 405.1180 | -1.48 | 390.0935[M+H-CH_3_]^+^,  375.0709[M+H-2CH_3_]^+^,  372.0864[M+H-CH_3_-H_2_O]^+^,  357.0603[M+H-2CH_3_-H_2_O]^+^,  347.0759[M+H-2CH_3_-CO]^+^,  332.0528[M+H-3CH_3_-CO]^+^,  329.0665[M+H-2CH_3_-H_2_O-CO]^+^,  317.0280[M+H-4CH_3_-CO]^+^,  304.0570[M+H-3CH_3_-2CO]^+^,  301.0710[M+H-2CH_3_-H_2_O-2CO]^+^,  289.0342[M+H-4CH_3_-2CO]^+^ | 5,4´-Dihydroxy-3,6,7,8,3´-pentamethoxyflavone | Flavonol |
| 174 | 23.80 | C_20_H_20_O_8_ | 389.1228 | -2.06 | 374.1007[M+H-CH_3_]^+^,  359.0764[M+H-2CH_3_]^+^,  356.0887[M+H-CH_3_-H_2_O]^+^,  341.0659[M+H-2CH_3_-H_2_O]^+^,  331.0800[M+H-2CH_3_-CO]^+^,  328.0940[M+H-CH_3_-H_2_O-CO]^+^,  313.0706[M+H-2CH_3_-H_2_O-CO]^+^ | [5-Demethylnobiletin](https://www.chemsrc.com/cas/2174-59-6_964689.html) *^a^* [11] | Flavone |
| 175 | 23.93 | C_21_H_22_O_8_ | 403.1384 | -2.23 | 388.1154[M+H-CH_3_]^+^,  373.0917[M+H-2CH_3_]^+^,  355.0817[M+H-2CH_3_-H_2_O]^+^,  327.0871[M+H-2CH_3_-H_2_O-CO]^+^ | Quercetagetin-3,5,6,7,3′,4′-hexamethyl ether isomer/[Nobiletin](https://www.chemsrc.com/cas/478-01-3_122104.html) isomer | Flavonol |
| 176 | 24.07 | [C](https://pubchem.ncbi.nlm.nih.gov/#query=C20H22O7)_20_H_22_O_7_ | 375.1440 | -1.07 | 211.0602[M+H-C_10_H_12_O_2_]^+^,  196.0365[M+H-C_10_H_12_O_2_-CH_3_]^+^,  191.0697[M+H-C_9_H_12_O_4_]^+^,  150.0318[M+H-C_10_H_12_O_2_-CH_3_-CO-H_2_O]^+^ | 2´-Hydroxy-3,4,4´,5´,6´-pentamethoxychalcone isomer | Chalcone |
| 177 | 24.13 | C_20_H_20_O_8_ | 389.1227 | -2.31 | 374.0997[M+H-CH_3_]^+^,  359.0763[M+H-2CH_3_]^+^,  341.0658[M+H-2CH_3_-H_2_O]^+^,  331.0821[M+H-2CH_3_-CO]^+^,  313.0711[M+H-2CH_3_-H_2_O-CO]^+^ | [5-Demethylnobiletin](https://www.chemsrc.com/cas/2174-59-6_964689.html) isomer | Flavone |
| 178 | 24.47 | C_20_H_20_O_8_ | 389.1228 | -2.06 | 374.1000[M+H-CH_3_]^+^,  359.0763[M+H-2CH_3_]^+^,  356.0886[M+H-CH_3_-H_2_O]^+^,  341.0649[M+H-2CH_3_-H_2_O]^+^,  331.0810[M+H-2CH_3_-CO]^+^,  328.0920[M+H-CH_3_-H_2_O-CO]^+^,  313.0713[M+H-2CH_3_-H_2_O-CO]^+^ | [5-Demethylnobiletin](https://www.chemsrc.com/cas/2174-59-6_964689.html) isomer | Flavone |
| 179 | 24.51 | C_21_H_24_O_8_ | 405.1541 | -1.97 | 241.0709[M+H-C_10_H_12_O_2_]^-^,  226.0472[M+H-C_10_H_12_O_2_-CH_3_]^+^,  211.0238[M+H-C_10_H_12_O_2_-2CH_3_]^+^,  208.0373[M+H-C_10_H_12_O_2_-CH_3_-H_2_O]^+^,  198.0529[M+H-C_10_H_12_O_2_-CH_3_-CO]^+^,  191.0703[M+H-C_10_H_14_O_5_]^+^,  183.0288[M+H-C_10_H_12_O_2_-2CH_3_-CO]^+^ | 2´-Hydroxy-3,4,3´,4´,5´,6´-hextamethoxychalcone isomer | Chalcone |
| 180 | 24.61 | C_21_H_22_O_9_ | 419.1333 | -2.15 | 404.1103[M+H-CH_3_]^+^,  389.0869[M+H-2CH_3_]^+^,  386.1012[M+H-CH_3_-H_2_O]^+^,  374.0627[M+H-3CH_3_]^+^,  371.0759[M+H-2CH_3_-H_2_O]^+^,  361.0914[M+H-2CH_3_-CO]^+^,  346.0682[M+H-3CH_3_-CO]^+^,  343.0814[M+H-2CH_3_-CO-H_2_O]^+^,  331.0448[M+H-4CH_3_-CO]^+^,  328.0590[M+H-3CH_3_-CO-H_2_O]^+^,  313.0317[M+H-4CH_3_-CO-H_2_O]^+^ | 8-Hydroxy-3,5,6,7,3′,4′-hexamethoxyflavone *^a^* | Flavonol |
| 181 | 25.04 | C_19_H_18_O_7_ | 359.1125 | -1.67 | 344.0889[M+H-CH_3_]^+^,  329.0656[M+H-2CH_3_]^+^,  326.0751[M+H-CH_3_-H_2_O]^+^,  298.0847[M+H-CH_3_-H_2_O-CO]^+^,  283.0603[M+H-2CH_3_-H_2_O-CO]^+^ | 5-Hydroxy-3,7,3',4'-tetramethoxyflavone *^a^* | Flavonol |
| 182 | 25.26 | [C](https://pubchem.ncbi.nlm.nih.gov/#query=C20H22O7)_20_H_22_O_7_ | 375.1439 | -1.33 | 211.0599[M+H-C_10_H_12_O_2_]^+^,  196.0361[M+H-C_10_H_12_O_2_-CH_3_]^+^,  191.0698[M+H-C_9_H_12_O_4_]^+^,  168.0417[M+H-C_10_H_12_O_2_-CH_3_-CO]^+^,  150.0309[M+H-C_10_H_12_O_2_-CH_3_-CO-H_2_O]^+^ | 2´-Hydroxy-3,4,4´,5´,6´-pentamethoxychalcone isomer | Chalcone |
| 183 | 25.47 | C_19_H_18_O_7_ | 359.1125 | -1.67 | 344.0893[M+H-CH_3_]^+^,  329.0660[M+H-2CH_3_]^+^,  326.0787[M+H-CH_3_-H_2_O]^+^,  298.0829[M+H-CH_3_-H_2_O-CO]^+^,  283.0597[M+H-2CH_3_-H_2_O-CO]^+^ | 5-Hydroxy-3,7,3',4'-tetramethoxyflavone isomer | Flavonol |
| 184 | 25.48 | C_19_H_20_O_6_ | 345.1335 | -0.87 | 211.0598[M+H-C_9_H_10_O]^+^,  196.0362[M+H-C_9_H_10_O-CH_3_]^+^,  150.0312[M+H-C_9_H_10_O-CH_3_-CO-H_2_O]^+^ | [5,6,7,4'-Tetramethoxyflavanone](https://www.chemsrc.com/cas/72943-90-9_1680019.html) isomer | Dihydroflavonoid |
| 185 | 25.71 | C_19_H_20_O_6_ | 345.1333 | -1.45 | 211.0595[M+H-C_9_H_10_O]^+^,  196.0360[M+H-C_9_H_10_O-CH_3_]^+^,  150.0310[M+H-C_9_H_10_O-CH_3_-CO-H_2_O]^+^ | [5,6,7,4'-Tetramethoxyflavanone](https://www.chemsrc.com/cas/72943-90-9_1680019.html) isomer | Dihydroflavonoid |
| 186 | 25.87 | C_20_H_20_O_8_ | 389.1230 | -1.54 | 374.0988[M+H-CH_3_]^+^,  359.0758[M+H-2CH_3_]^+^,  356.0878[M+H-CH_3_-H_2_O]^+^,  341.0651[M+H-2CH_3_-H_2_O]^+^,  331.0808[M+H-2CH_3_-CO]^+^,  328.0953[M+H-CH_3_-H_2_O-CO]^+^,  313.0710[M+H-2CH_3_-H_2_O-CO]^+^ | [5-Demethylnobiletin](https://www.chemsrc.com/cas/2174-59-6_964689.html) isomer | Flavone |
| 187 | 26.61 | C_19_H_20_O_6_ | 345.1334 | -1.16 | 211.0599[M+H-C_9_H_10_O]^+^,  196.0365[M+H-C_9_H_10_O-CH_3_]^+^,  150.0311[M+H-C_9_H_10_O-CH_3_-CO-H_2_O]^+^ | [5,6,7,4'-Tetramethoxyflavanone](https://www.chemsrc.com/cas/72943-90-9_1680019.html) isomer | Dihydroflavonoid |

*a*: Compound confirmed by comparison with the reference standard.

References

1. Zhang, K. *et al.* Chemome profiling of *Citri Ｒeticulatae Pericarpium* using UHPLC-IT-TOF-MS.*Chin. J. Chin. Mater. Med*. **45**, 899-909 (2020).
2. Zhu, S.X., Zhang, J.Y., Lv, Z.H. & Yu, M.M. LC-MS/MS determination of apigenin in rat plasma and application to pharmacokinetic study. *Curr. Pharm. Biotechnol*. **22**, 274-280 (2021)
3. Mohott, S. *et al.* Screening for bioactive secondary metabolites in Sri Lankan medicinal plants by microfractionation and targeted isolation of antimicrobial flavonoids from Derris scandens. *J. Ethnopharmacol*. **246**, 112158. (2020).
4. Wang, P. *et al*. Chemical and genetic discrimination of commercial Guangchenpi (Citrus reticulata 'Chachi') by using UPLC-QTOF-MS/MS based metabolomics and DNA barcoding approaches. *RSC Adv*. **9**, 23373-23381 (2019).
5. Maharani, A. A. *et al*. LC-QTOF-MS/MS Based Molecular Networking Approach for the Isolation of α-Glucosidase Inhibitors and Virucidal Agents from Coccinia grandis (L.) Voigt. *Foods* **10**, 3041(2021).
6. Braunberger, C. *et al*. LC-NMR, NMR, and LC-MS identification and LC-DAD quantification of flavonoids and ellagic acid derivatives in Drosera peltata. *J. Chromatogr. B Analyt. Technol. Biomed. Life Sci*. **932**, 111-116 (2013).
7. [Bianchi](https://pubmed.ncbi.nlm.nih.gov/?term=Bianchi+M&cauthor_id=29258044), M., [Canavesi](https://pubmed.ncbi.nlm.nih.gov/?term=Canavesi+R&cauthor_id=29258044), R., [Aprile](https://pubmed.ncbi.nlm.nih.gov/?term=Aprile+S&cauthor_id=29258044), S., [Grosa](https://pubmed.ncbi.nlm.nih.gov/?term=Grosa+G&cauthor_id=29258044), G. & Grosso, [E.D.](https://pubmed.ncbi.nlm.nih.gov/?term=Del+Grosso+E&cauthor_id=29258044) Troxerutin, a mixture of O-hydroxyethyl derivatives of the natural flavonoid rutin: Chemical stability and analytical aspects. *J. Pharm. Biomed. Anal*. **150**, 248-257 (2018).
8. Marzouk, M. *et al*. LC/HRESI-MS/MS screening, phytochemical characterization, and in vitro antioxidant and cytotoxic potential of Jatropha integerrima Jacq. extracts. *Bioorg. Chem*. **140**, 106825 (2023).
9. Kuźniewski, P., Załuski, D., Olech, M., Banaszczak, P. & Nowak, R. LC-ESI-MS/MS profiling of phenolics in the leaves of Eleutherococcus senticosus cultivated in the West Europe and anti-hyaluronidase and anti-acetylcholinestarase activities. *Nat. Prod. Res.* **32**, 448-452 (2018).
10. Choudhury, P. *et al*. Assessment of nutritional value and quantitative analysis of bioactive phytochemicals through targeted LC-MS/MS method in selected scented and pigmented rice varietals. *J. Food Sci*. **85**, 1781-1792 (2020).
11. Zheng, J.K. *et al*. Identification of novel bioactive metabolites of 5-demethylnobiletin in mice. *Mol. Nutr. Food Res*. **57**, 1999-2007 (2013).

Fig. S1 The LC-MS traces of flavonoids on total ion chromatogram of *Citri Reticulatae Pericarpium* (A-E).
